# An ester bond underlies the mechanical strength of a pathogen surface protein

**DOI:** 10.1038/s41467-021-25425-6

**Published:** 2021-08-23

**Authors:** Hai Lei, Quan Ma, Wenfei Li, Jing Wen, Haibo Ma, Meng Qin, Wei Wang, Yi Cao

**Affiliations:** 1 Collaborative Innovation Center of Advanced Microstructures, National Laboratory of Solid State Microstructure, Department of Physics, Nanjing University, Nanjing, China; 2 Chemistry and Biomedicine Innovation Center, Nanjing University, Nanjing, China; 3 Key Laboratory of Mesoscopic Chemistry of MOE, School of Chemistry and Chemical Engineering, Institute of Theoretical and Computational Chemistry, Nanjing University, Nanjing, China

**Keywords:** Protein folding, Single-molecule biophysics, Bacterial adhesion, Atomic force microscopy

## Abstract

Gram-positive bacteria can resist large mechanical perturbations during their invasion and colonization by secreting various surface proteins with intramolecular isopeptide or ester bonds. Compared to isopeptide bonds, ester bonds are prone to hydrolysis. It remains elusive whether ester bonds can completely block mechanical extension similarly to isopeptide bonds, or whether ester bonds dissipate mechanical energy by bond rupture. Here, we show that an ester-bond containing stalk domain of Cpe0147 is inextensible even at forces > 2 nN. The ester bond locks the structure to a partially unfolded conformation, in which the ester bond remains largely water inaccessible. This allows the ester bond to withstand considerable mechanical forces and in turn prevent complete protein unfolding. However, the protecting effect might be reduced at non-physiological basic pHs or low calcium concentrations due to destabilizing the protein structures. Inspired by this design principle, we engineer a disulfide mutant resistant to mechanical unfolding under reducing conditions.

## Abstract (web summary)

Bacterial surface adhesion proteins are characterized by unusual mechanical properties. Here, the authors use atomic force microscopy-based technique to study a surface-anchoring protein Cpe0147 from *Clostridium perfringens* and show that an ester bond can withstand considerable mechanical forces and prevent complete protein unfolding.

## Introduction

Gram-positive bacteria produce a variety of surface adhesion proteins for host surface binding, biofilm formation, and immune evasion^1–6^. These proteins include rod-like pili and other large multidomain proteins such as microbial surface components recognizing adhesive matrix molecules (MSCRAMMs)^7,8^. Despite that these proteins have distinct synthesis pathways, they share similar ‘beads on a string’ extended organization. Many of the domains in these proteins adopt Ig-like structure with unusual intramolecular covalent cross-links^9,10^, including thioester^11–13^, isopeptide^14,15^, and ester bonds^16^. These bonds endow the proteins outstanding mechanical functions for strong surface anchoring or resisting large mechanical perturbations. Depending on the chemical reactivity and the location in the protein structures, thioester and isopeptide bonds have distinct mechanical functions. Isopeptide bonds are generally formed between the side chains of Lys and Asn (or Asp) residues and are chemically inert under physiological conditions^9,17–20^. Both single-molecule force spectroscopy experiments^15,21,22^ and molecular dynamics simulations^23^ revealed that isopeptide bond containing proteins can withstand considerable mechanical forces. For example, spy0128, the major pilin subunit of *Streptococcus pyogenes*, is inextensible up to 800 pN due to the presence of isopeptide bond between the first and the last β strands^15^. Moreover, as the isopeptide bond is located at the site of stress concentration, it locks the protein in a partially unfolded structure and allows fast refolding under load^23^. In another pilin subunit SpaA from *Corynebacterium diphtheriae*, the isopeptide bond can also restrict the unfolding of this CnaA Ig-type domain from complete unfolding under mechanical stress^22^. Unlike isopeptide bonds, thioester bonds are more labile and can react with amino groups that are abundant in host tissues^12^. They are often present at pilin tip-end adhesins and are formed in between the side chains of Cys and Gln residues. The reaction of thioester with nucleophilic ligands creates strong covalent adhesion with the host surfaces to resist large mechanical stresses^12^. Interestingly, recent studies revealed that the reactivity of thioester bonds is high at low forces and can be completely blocked at forces larger than 35 pN, entailing the bacteria stress dependent mobility^24^.

Ester bond is another type of intramolecular covalent bonds discovered in Gram-positive surface proteins and is formed between the side chains of Thr and Gln residues^9,16,25^. It was first discovered in a surface-anchoring protein Cpe0147 from *Clostridium perfringens* and then widely found in many bacterial surface adhesion molecules. The ester bonds in those proteins generally locate at the same position as the isopeptide bond in spy0128. However, as ester bonds are prone to hydrolyze at both acidic and basic conditions^26^, it is largely unknown how such unique chemical properties affect the mechanical properties of ester bond containing proteins. Previous studies showed that Cpe0147 is resistant to hydrolysis under normal physiological conditions, implying that the ester bond is shielded from the attack of OH^−^ ions^25^. However, as mechanical force may locally destabilize the protein structures and then expose the buried ester bond to water, it remains elusive whether the ester bond containing proteins have distinct mechanical responses compared to the isopeptide bond containing proteins. Revealing the mechanical response of the ester bond containing proteins may also provide new insights into the pathological mechanism of Gram-positive bacteria and help to develop antibiotics based on blocking the formation of ester bonds.

In this work, we employ atomic force microscopy (AFM)-based single-molecule force microscopy^27–32^, protein engineering, and molecular dynamics simulation to study the mechanical properties of the C1 domain of Cpe0147. We find that C1 exhibits similar mechanical stability as the isopeptide bond containing protein spy0128 and is inextensible even under forces > 2 nN. In contrast, if force is applied from a different orientation to unfold the protein structure, the ester bond breaks at forces of only ~80 pN. These results suggest that the interplay between the ester bond and the protein structure is critical to the mechanical properties of the C1 domain. Molecular dynamics simulations reveal that the ester bond locks the structure to a partially unfolded conformation, in which the ester bond remains largely water inaccessible. This allows the ester bond to withstand considerable mechanical forces and in turn to prevent complete protein unfolding. Such a coupling between the ester bond and the protein structure is also found in another ester containing proteins, ParV of *Parvimonas sp*. Even when the ester bond is replaced by a disulfide, the mechanical stability of the protein is not affected. On the other hand, once the structure of the protein is destabilized at a basic pH or low calcium conditions, which are usually found on wound surfaces, some of the C1 domains can be unfolded, leading to the hydrolysis of the ester bond under mechanical load. Taken together, our studies reveal an uncharted mechanism to allow adhesion proteins to withstand high mechanical load using hydrolysable ester bonds. The environment-dependent mechanical stability may be related to the biological functions of Cpe0147 in vivo.

## Results

### Ester bond containing C1 domain is inextensible under mechanical forces > 2 nN

**Fig. 1:**
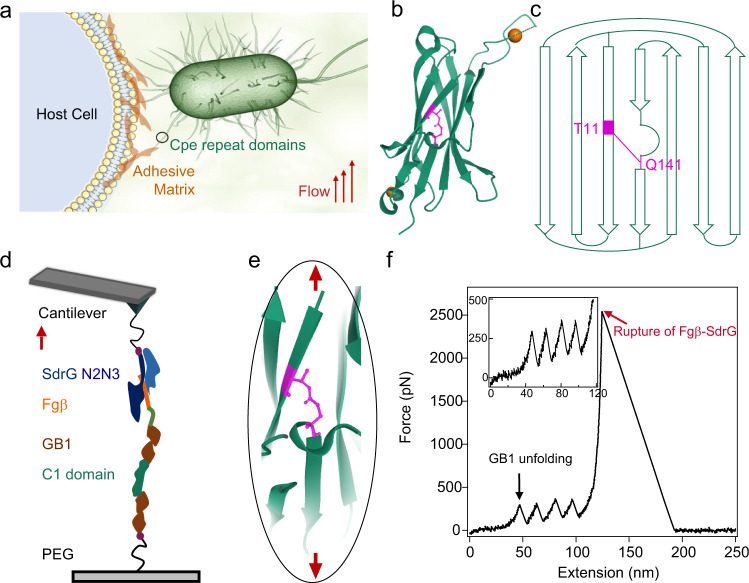
Cpe0147 is highly resistant to mechanical forces. **a** Cpe0147 links the tip adhesion domain and bacteria to establish invasion and colonization and experiences considerable mechanical forces in the biological settings. **b** Structure of the C1 domain from PDB (4MKM). The ester bond is highlighted in magenta and the two calcium ions are shown as orange spheres. **c** Topology of C1_*WT*_ domain. The ester bond is formed between Thr-11 and Gln-141. **d** Schematic of the AFM-based single-molecule force spectroscopy experiments. Fgβ-(GB1)_2_-C1_*WT*_-(GB1)_2_-cys was covalently anchored to a glass surface through a polyethylene glycol (PEG) linker via thiol–maleimide chemistry and picked up using a SdrG-cys modified cantilever. **e** Mechanical extension of C1_*WT*_ from its N- and C-termini is blocked by the ester bond as the ester bond is located right at the force concentration point. **f** A representative single-molecule force–extension trace at 1.6 μm s^−1^ showing the unfolding of the four GB1 domains (black arrow) at ~200 pN but no unfolding peak of C1_*WT*_ up to the rupture forces of Fgβ-SdrG complexes (red arrow). This experiment was repeated more than six times independently with similar results. Source data are provided as a Source Data file.

Cpe0147 is a multiple-domain protein that covalently links the bacterial cell wall and the adhesin^16^ (Fig. 1a). The domains in the stalk region adopt similar all β-strand IgG-like fold and contains an ester bond linked the Thr on the first and the Gln on the last β-strands^16^ (Fig. 1b, c). To investigate the mechanical response of Cpe0147, we used the wide type C1 domain (C1_*WT*_) as the model system. We engineered a chimeric polyprotein, Fgβ-(GB1)_2_-C1_*WT*_-(GB1)_2_-cys, for single-molecule AFM experiments (Fig. 1d), following the experimental protocol reported by Yu et al.^27^. This polyprotein features specific noncovalent/covalent linkage of the polyprotein to either the cantilever tip or the substrate as well as mechanical fingerprint units for unambiguously identifying single-molecule events. The proteins were covalently linked to the substrate through the thiol group of the C-terminal Cys and then picked up by the SdrG modified cantilever tip through strong noncovalent interactions between Fgβ and SdrG (rupture forces >2 nN)^28,33^. Each end of C1_*WT*_ was flanked with two GB1 domains. The mechanical unfolding of GB1 was characterized by a contour length increment of ~18 nm and an unfolding force of ~200 pN at a pulling speed of 1.6 μm s^−1^^34–36^. Stretching the polyprotein allowed us to apply forces to C1_*WT*_ between its N- and C-termini, similar to the force direction the protein domain experiences in their biological settings.

Stretching polyprotein led to representative sawtooth-like force–extension curves (Fig. 1f, Supplementary Fig. 1), in which each sawtooth peak corresponds to the force-induced unfolding of an individual domain in the polyprotein chain. However, we only observed five peaks; four of them showed the same contour length increments of 18 nm, which can be attributed to the unfolding of the four GB1 domains. The last peak of more than 2 nN arisen from the unbinding of the Fgβ/SdrG interaction^28^. There were no other peaks observed and the contour length of the last peak is about 130 nm (Supplementary Fig. 2) indicating that C1_*WT*_ did not fully unfold even under pulling forces higher than 2 nN. Note that, we observed a few events (24 in 805) with the first peak appeared at much longer contour length than normal ones, which may correspond to the proteins with the ester bond containing domain fully unfolded before stretching (Supplementary Table 1). Increasing the pulling speed can increase the rupture forces of the Fgβ/SdrG complexes. However, we still did not observe the rupture of ester bonds (Supplementary Table 2 and Supplementary Fig. 3). We intended to further increase the maximum forces applied to C1_*WT*_, by using covalent linkages to anchor the polyproteins to both the cantilever tip and the substrate. We constructed another polyprotein, Spytag-(GB1-C1_*WT*_)_4_-cys. Spytag can bind Spycatcher to form a covalent isopeptide bond^37,38^. Unfortunately, the maximum detaching force in this design was even lower than that based on the Fgβ/SdrG interaction, presumably due to the maleimide–thiol chemistry we used for protein immobilization, which was not strong enough if the conjugate was not hydrolyzed^39^ (Supplementary Note 1). In this covalent linking scheme, each molecule can be stretched only once, preventing the mechanically triggered hydrolysis of maleimide–thiol adducts^39^ (Supplementary Note 2). Nonetheless, we only observed the unfolding events from four GB1 domains using this polyprotein (Supplementary Fig. 4). As the formation of intramolecular ester bond in the C1 domain was further confirmed by using the mass spectroscopy (Supplementary Fig. 5), it is safe to conclude that the wild-type C1 domain from Cpe0147 is inextensible up to the forces that can break weak covalent bonds.

### Mechanical stability of the C1 domain without ester bond

**Fig. 2:**
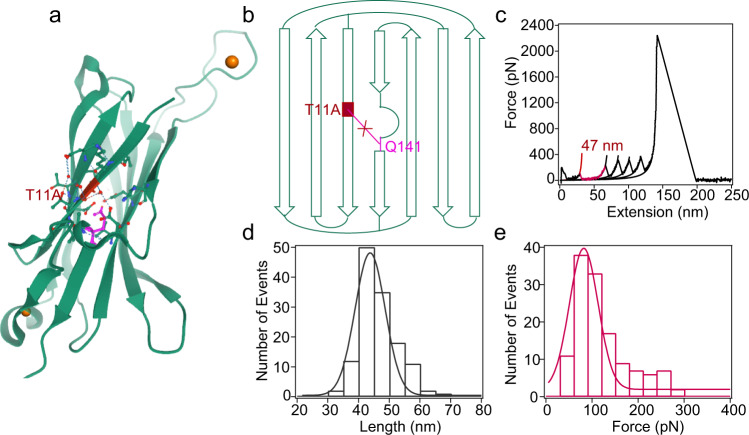
Mechanical unfolding of the mutated C1 domain without the ester bond. **a** Thr-11 of C1 was mutated to Ala to eliminate the ester bond to yield the C1_*T11A*_ mutant. **b** Topology of C1_*T11A*_. The ester bond cannot form in the mutant. **c** Representative single-molecule force–extension curve of stretching Fgβ-(GB1)_2_-C1_*T11A*_-(GB1)_2_-cys following the same experimental protocol shown in Fig. 1d. Each peak was fitted by worm-like chain (WLC) model of polymer elasticity. The peak with a Δ*L*_*c*_ of 47 nm corresponds to the unfolding of C1_*T11A*_, the next four peaks correspond to the unfolding of GB1 domains, and the last peak corresponds to the rupture of the Fgβ/SdrG complex. **d** Histogram of contour length increment for C1 unfolding is centered ~47 nm. **e** Unfolding force histogram of C1 domain at a pulling speed of 1.6 μm s^−1^ measures an average unfolding force of 92 ± 41 pN (*n* = 129, total number of C1 unfolding events). Fitting the force distribution with Monte Carlo simulation^65^ results in a Δx_u_ of 0.27 nm and α_0_ of 0.35 s^−1^ (Supplementary Fig. 8). This experiment was repeated three times independently with similar results. Source data are provided as a Source Data file.

To illustrate the role of ester bond in maintaining high mechanical stability of the C1 domain, we designed a protein variant C1_*T11A*_ in which the ester bond was eliminated by mutating ester bond forming Thr at the 11th position to Ala (Fig. 2a, b) (Supplementary Fig. 6). Also, the polyprotein Fgβ-(GB1)_2_-C1_*T11A*_-(GB1)_2_-cys was constructed and then studied using AFM-based single-molecule force spectroscopy. In the force–extension curves, besides the four peaks of Δ*L*_*c*_ of ~18 nm for GB1, we observed an additional peak of Δ*L*_*c*_ of 47.4 ± 3.25 nm (colored in red) (Fig. 2c, Supplementary Fig. 7). The contour length increment is consistent with the complete mechanical unfolding of C1 (Δ*L*_*c*_ = 146 aa × 0.365 nm/aa − 5.2 nm). The unfolding forces of C1_*T11A*_ domain were about 92 ± 41 pN (Fig. 2e), which were lower than that of GB1. Eliminating the ester bond by the T11A mutation significantly reduced the unfolding forces of C1_*WT*_. Therefore, the ester bond plays an important role in stabilizing the wild-type C1 structure under mechanical load.

### Ester bond is mechanically weak in the absence of the folded C1 structure

**Fig. 3:**
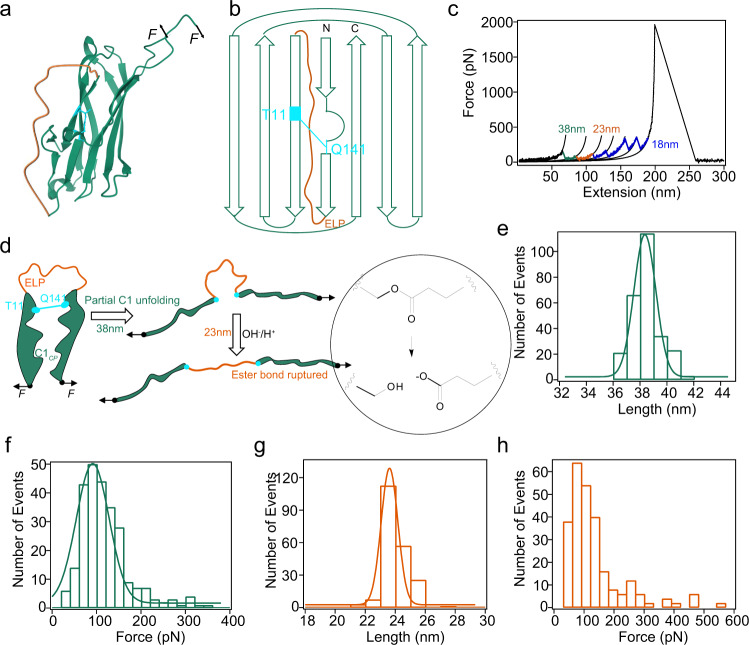
Mechanical unfolding of the circular permutant of C1, C1_*CP*_. **a** Structure of C1_*CP*_ based on PDB (4MKM). **b** Topology of C1_*CP*_. Ester bond formed between T11 and Q141 is highlighted in cyan. The new N- and C-termini are at the position 126 and 125, respectively. The original N- and C-termini are connected by an ELP loop. **c** Representative force–extension curve of stretching (GB1)_2_-C1_*CP*_-(GB1)_2_ following the same experimental protocol shown in Fig. 1d. The force peaks with Δ*L*_*c*_ of ~38 nm (green) and ~23 nm (orange) are assigned as the unfolding of C1_*CP*_ and the rupture of ester bond, respectively. The force peaks of Δ*L*_*c*_ of ~18 nm correspond to the unfolding of GB1 domains. **d** Schematic illustration of the contour length change upon stretching C1_*CP*_. First, the protein unfolds up to the ester bond position, then the rupture of ester bond releases the sequestered sequence. **e** Histogram of Δ*L*_*c*_ for C1_*CP*_ unfolding events peaks at 38 nm. **f** Unfolding force histogram of C1_*CP*_ centers at 91.5 ± 52 pN (*n* = 243, total number of C1 unfolding events). The Monte Carlo simulation of the force distribution results in a Δx_u_ of 0.25 nm and α_0_ of 0.37 s^−1^ (Supplementary Fig. 14). **g** Histogram of Δ*L*_*c*_ corresponding to the rupture of ester bond in C1_*CP*_ peaks at 23 nm. **h** Histogram of the rupture forces of ester bond centers at 77 ± 56 pN (*n* = 243, total number of rupture of ester bond events). This experiment was repeated three times independently with similar results. Source data are provided as a Source Data file.

To further understand the contribution of ester bond to the mechanical stability of C1, we design a circular permutant of C1 (C1_*CP*_) to structurally decouple the rupture of ester bond and the unfolding of C1_*WT*_. In C1_*CP*_, the N- and C-termini of C1 were connected by an elastin-like peptide (ELP) of 45 amino acids, and the protein was split between the residues 125 and 126 to form the new N- and C-termini (Fig. 3a, b). Previously, Young et al. have shown that the two fragments obtained by splitting C1_*WT*_ at this position can spontaneous rebind and form the inter-molecular ester bond^25^. Therefore, we anticipated that the ester bond can also formed in C1_*CP*_. Mass spectroscopy analysis of C1_*CP*_ confirmed the formation of the ester bond (Supplementary Fig. 9). Note that in this new pulling direction, the ester bond is not located at the force concentration point (the position in the protein where the force is significantly higher than surrounding region) anymore. We would expect to see the rupture of ester bond after the unfolding of C1_*CP*_, providing an unambiguously way to quantify the strength of the ester bond in the absence of the folded protein structure.

C1_*CP*_ was stretched in the polyprotein Fgβ-(GB1)_2_-C1_*CP*_-(GB1)_2_-cys construct and the representative force–extension curve is shown in Fig. 3c and Supplementary Fig. 10. In addition to the four peaks whose Δ*L*_*c*_ correspond to the unfolding of GB1, we observed two additional peaks of Δ*L*_*c*_ of 38 ± 1.2 nm (colored in green) and 23 ± 0.5 nm (colored in orange). The event in green always occurred first and then followed by the event in orange. Matching to the protein structure, these two events can be assigned to the piecewise extension of C1_*CP*_ (Fig. 3d). The first peak corresponded to the unfolding of the protein and then extending the peptide sequence outside the ester bond (Thr-Gln). The measured Δ*L*_*c*_ matches well with the theoretically calculated value of ~39 nm (((110–12) + (140–129)) aa × 0.365 nm/aa – 0.9 nm + 0.6 nm), where 0.9 nm is the distance between V113 and Q129 and 0.6 nm is the length of the ester bond containing linkage between T11 and Q141 (Fig. 3e). The unfolding forces of C1_*CP*_ were 91.6 ± 52 pN (Fig. 3f). The second peak resulted from the force-induced rupture of the ester bond and subsequently extending the peptide sequence shielded by this bond, as the measured Δ*L*_*c*_ of 23 ± 0.5 nm matched well with the theoretically calculated value of 23 nm (66 aa × 0.365 nm/aa – 0.6 nm) (Fig. 3g). Moreover, the 23-nm peak was absent in the force–extension curves of the polyprotein containing C1_*CP*_-T11A, in which Thr was mutated to Ala to eliminate the ester bond (Supplementary Figs. 11 and 12). This further demonstrated that the peaks of Δ*L*_*c*_ of ~23 nm were indeed from the rupture of the ester bond in C1_*CP*_.The forces for the rupture of the ester bond were only 77 ± 56 pN (Fig. 3h), much lower than that of typical covalent bonds^40–43^ and even some noncovalent bonds^44–47^. We inferred that this may be because the rupture of ester bond in water undergoes a hydrolysis mechanism^26^, distinct from the free radical homolysis mechanism for the rupture of other covalent bonds^48^. As indicated by the theoretical calculation by Akbulatov et al.^26^, ester hydrolysis under stretching follows a two-step kinetics. The first step involves the attack of the nucleophile (OH^−^/H+) to form a tetrahedral intermediate and the second step corresponds to the decomposition of the C–O bond under force. The first step is typically the rate limiting step and shows a weak force-dependency with a free energy barrier within 1 kcal mol^−1^ based on the Bell-Evans model. The detailed ester hydrolysis mechanism under force may deserve further detailed experimental characterization, which is beyond the topic of this work. By repeatedly stretching and relaxing Fgβ-(GB1)_2_-C1_*CP*_-(GB1)_2_-cys, we were able to observe the reformation of the ester bond after rupture, suggesting that the ester bond rupture and formation are reversible under forces (Supplementary Fig. 13). These results clearly indicate that an unprotected ester bond is mechanically weak and the topology of the wild-type C1 structure is critical to its high mechanical stability.

### Molecular dynamics simulations

**Fig. 4:**
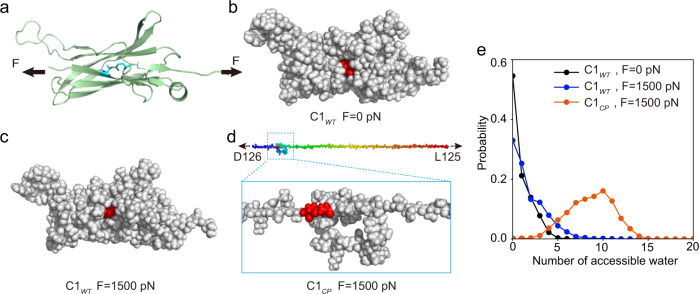
Molecular dynamics simulations demonstrating different water accessibility of the ester bond in C1_*WT*_ and C1_*CP*_ under pulling force. **a** Cartoon representation of the three-dimensional structure of C1_*WT*_ under the pulling force of 1500 pN. The residues Thr-11 and Gln-141 forming the ester bond were also shown by sticks representation. Three-dimensional structure of C1_*WT*_ shown by sphere representation without force (**b**) and under the pulling force of 1500 pN (**c**). **d** Three-dimensional structure of C1_*CP*_ under the pulling force of 1500 pN shown by sphere representation. For clarity, the zoom-in structure was also shown. The residues Thr-11 and Gln-141 were colored in red in **b**–**d**. **e** Distribution of the number of water molecules within 5 Å from the Oε1 atom of the Gln-141 for C1_*WT*_ (blue) and C1_*CP*_ (orange) at the constant pulling force of 1500 pN. For comparison, the result for C1_*WT*_ (black) without applying force was also shown. Source data are provided as a Source Data file.

To understand the molecular details underlying the ultrahigh mechanical stability of C1, we performed steered molecular dynamics simulations for both C1_*WT*_ and C1_*CP*_ (Fig. 4a) (see “Methods” for more details). For C1_*WT*_ without force load, the ester bond was buried to a large extent and shielded from water attack by the nearby residues (Fig. 4b). When a constant pulling force of 1500 pN was applied to the N- and C-terminal residues, the protein structure only partially unfolded at the force bearing first and last β-strands. However, as the ester bond was located at the force-concentrating point, it prevented force propagation to the rest parts of the protein and made the partially unfolded structure resemble the native structure (Fig. 4c). As the rupture of the ester bond requires water attack, the structural integrity of C1_*WT*_ can in turn ensure high mechanical stability of the ester bond. In comparison, for C1_*CP*_, the ester bond started to sustain the pulling force only at the later stage of the unfolding event. Applying pulling force led to complete protein unfolding and exposure of the ester bond to water (Fig. 4d). To estimate the reactivity of the ester bonds in the three conditions, we quantified the number of water molecules within 5 Å from the Oε1 atom of the Gln-141. For C1_*WT*_, even at a constant pulling force of 1.5 nN, the number of water molecules accessible to the ester bond was only slightly higher than that without force load (Fig. 4e). In contrast, for C1_*CP*_, the ester bond was surrounded by ~9 water molecules due to the unfolding of the protein structure. Detailed structural analysis revealed that the bulky side chains of residues K10, T12, H133, D138, and A140 near the ester bond form a cage to shield the water molecules outside (Supplementary Fig. 15a). This cage structure was well maintained in C1_*WT*_ even under a stretching force of 1.5 nN and only slightly open at 2 nN, as indicated by the root-mean-square deviation (RMSD) of these residues (Supplementary Fig. 15b). However, in C1_*CP*_, this cage fell apart quickly, making the ester bond exposed to water molecules. Moreover, without the ester bond, even in C1_*WT*_, the residues that form the ester bond were exposed to water molecules within a few hundreds of picoseconds (Supplementary Fig. 15c). The maximum number of water molecules was ~14, which was even higher than that for C1_*CP*_ with the ester bond under forces. Comparing the distance evolution between the ester bond forming residues (Supplementary Fig. 15d) and the overall structural change of the protein in the absence of ester bond (Supplementary Figs. 15e and 16), we can clearly see that the separation of the ester bond forming residues was always prior to the increase of RMSD, indicating that breaking of the ester bond is a necessary step toward complete unfolding of the overall protein structure. Such results clearly demonstrated the mutual interplay between the chemical event and protein mechanical stability.

### C1 structure as a “safe house” to protect mechanical rupture of disulfide bonds

**Fig. 5:**
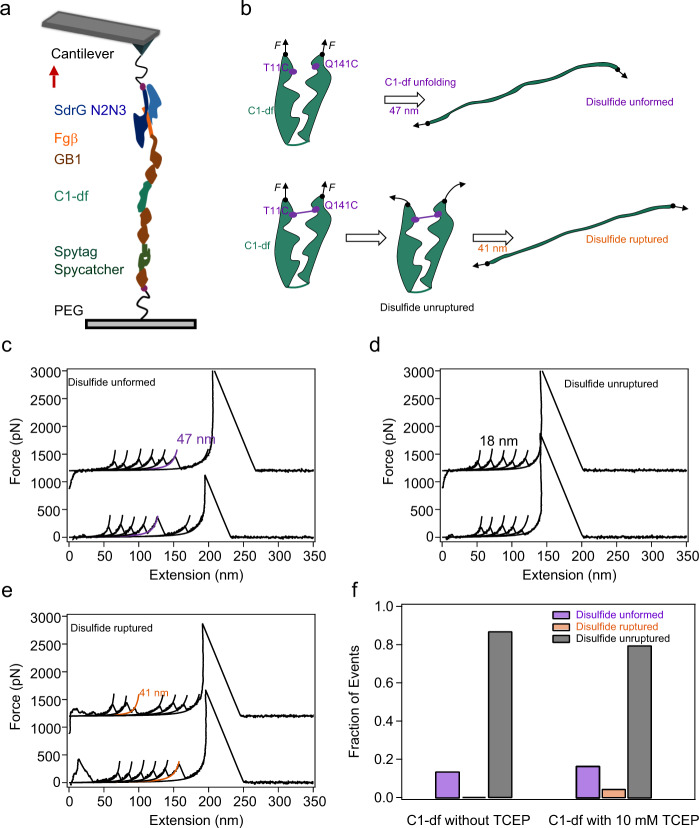
Mechanical unfolding of C1-df. **a** Schematic of the AFM-based single-molecule force spectroscopy experiments. Polyprotein Fgβ-(GB1)_2_-C1-df-(GB1)_2_-Spytag was linked to the cys-GB1-Spycatcher modified substrate covalently through the Spycatcher/Spytag chemistry and picked up by the SdrG-cys modified cantilever through the Fgβ/SdrG interaction. Thus, the force–extension curves should contain five GB1 fingerprints. **b** Three possible unfolding/hydrolysis pathways of C1-df under load and the corresponding contour length change. **c** Representative force–extension curves of the “Disulfide unformed” group. Two populations of unfolding events as showed, five peaks in black related to GB1 unfolding and the purple one with Δ*L*_*c*_ of 47 nm corresponds to the unfolding of C1-df. **d** Representative force–extension curves of the “Disulfide unruptured” group. The traces show only five GB1 unfolding events without the signature of C1-df unfolding or disulfide bond rupture. **e** Representative force–extension curves of the “Disulfide ruptured” group. Except for the five GB1 unfolding events, there is an additional peak with Δ*L*_*c*_ of 41 nm (colored in orange) corresponding to the rupture of the disulfide bond. **f** 2D bar shows the relative populations of the three kinds of events without (left, *n* = 193) or with (right, *n* = 389) 10 mM TCEP. This experiment was repeated 4 times independently with similar results. Source data are provided as a Source Data file.

**Fig. 6:**
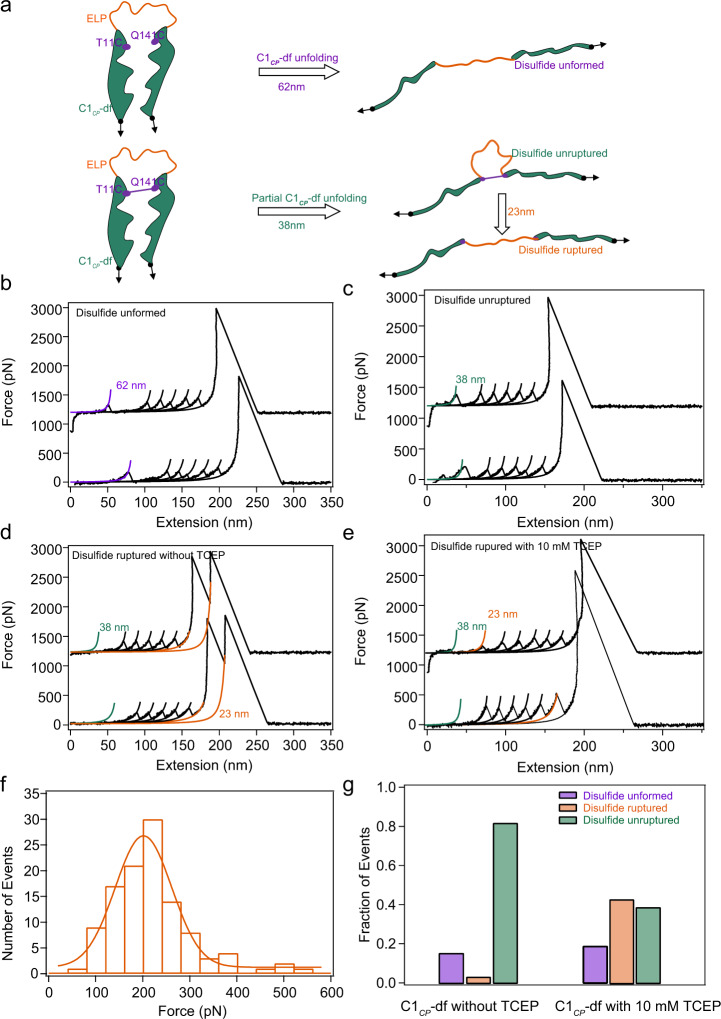
Mechanical unfolding of C1_*CP*_-df. C1_*CP*_-df was constructed in the same way as C1_*CP*_ except that the ester bond was replaced by a disulfide bond. **a** Three possible unfolding/hydrolysis pathways of C1_*CP*_-df under load and the corresponding contour length change. **b** Representative force–extension curves of the “Disulfide unformed” group. **c** Representative force–extension curves of the “Disulfide unruptured” group. Representative force–extension curves of the “Disulfide ruptured” group in PBS with (**d**) and without (**e**) 10 mM TCEP. **f** Histogram of the mechanical rupture force of disulfide bond in PBS with 10 mM TCEP. Fitting the force distribution with Monte Carlo simulation results in a Δx_u_ of 0.13 nm and _0_ of 0.39 s^−1^ (Supplementary Fig. 18). **g** 2D bars show the relative populations of the three kinds of events without (left, *n* = 844) or with 10 mM TCEP (right, *n* = 754). This experiment was repeated more than five times independently with similar results. Source data are provided as a Source Data file.

Inspired by the molecular mechanism that allows the integration of labile ester bond and weak protein fold to achieve remarkable mechanical stability, we hypothesized that it is also possible to use the C1 structure as a mechanically robust cage or “safe house” to protect other chemically active bonds from attack. To this extend, we replaced the ester bond in C1 to disulfide bond^49,50^ through T11C and Q141C mutation (the protein is named as C1-df hereafter), and studied the mechanical stability of this protein in the presence of reducing agent (10 mM tris (2-carboxyethyl) phosphine, TCEP, Supplementary Note 3). We built a polyprotein Fgβ-(GB1)_2_-C1-df-(GB1)_2_-Spytag and linked it to the cys-GB1-Spycatcher modified substrate by using Spycatcher/Spytag interaction instead of direct thiol–maleimide interaction to avoid the formation of mismatched disulfide bonds between the C-terminal Cys and Cys11/Cys141 that otherwise would complicate the single-molecule AFM experiments. The experimental scheme is shown in Fig. 5a. Note that the disulfide bond was not 100% formed in all proteins. Thus, the mechanical unfolding of C1-df could result in three different scenarios (Fig. 5b): (1) the unfolding of C1-df without the disulfide bond (denoted as the “Disulfide unformed” group); (2) the complete locking of C1-df with disulfide bond at the folded state without unfolding (denoted as the “Disulfide unruptured” group); and (3) the hydrolysis of the disulfide bond in C1-df and the release of the sequestered peptide sequences upon stretching (denoted as the “Disulfide ruptured” group). The representative force–extension curves of the three groups are shown in Fig. 5c–e (Supplementary Note 4). Unlike the case of ester bond, which forms in all C1 domains, the disulfide bond is only formed in 86.7% of the total events (168 out of 193) (Fig. 5f). Among them, the “Disulfide ruptured” events only account for 4.3% (16 out of 389) of the total events (Fig. 5f). However, in the absence of reducing agent, this group is missing. This indicates that the disulfide bond in C1-df is also protected from the attack of the reducing agent (Supplementary Fig. 17). To further confirm this, we studied the mechanical unfolding of the circular permutant of C1-df (C1_*CP*_-df), by replacing the ester bond in C1_*CP*_ to disulfide bond (Fig. 6). As in C1_*CP*_-df, the partially unfolded protein structure cannot prevent the attack from TCEP, the rupture of disulfide bonds was observed in 42.7% of the total events. Even without TECP, in 3.1% of the traces, we observed the rupture of disulfide bonds at forces of ~2 nN. Taken together, our results indicated that the C1 structure can efficiently prevent the reducing of the imbedded disulfide bond in the reducing environment under forces, similar to the protecting effect for the ester bond in C1_*WT*_. This contrasts with other disulfide bond containing proteins studied previously^49,51,52^. In those proteins, the disulfide bond was caged in folded protein structures and can be decaged by mechanical unfolding the proteins. Note that other reducing agents, such as L-glutathione hydrochloride, β-mercaptoethanol, and 1,4-DL-dithiothreitol, can also reduce the disulfide bonds. As they are of different size and reduce disulfide bonds following different mechanisms^53^, the shielding effect of the C1 structure for these reducing agents may be different from that for TCEP.

## Discussion

By employing single-molecule force spectroscopy, protein engineering, and molecular dynamics simulation, we studied the nanomechanical properties of the C1 domain of Cpe0147 from *Clostridium perfringens* with a spontaneously formed intramolecular ester bond. Our results revealed that C1 can retain a partially unfolded structure under a force as high as 2.5 nN. When the ester bond was removed, the mechanical stability of the protein C1_*T11A*_ dropped sharply with unfolding forces of ~92 pN. This demonstrates the important role of covalent ester bond for the mechanical stability of C1. However, using a circular permutation of C1 (C1_*CP*_), we revealed that the protein can first partially unfold at a force of ~90 pN and then rupture the ester bond at a force of ~80 pN. Although all intramolecular interactions are kept in C1_*CP*_, the change of the force orientation can dramatically affect the mechanical response. These results highlight the importance of the interplay of the protein structure and the ester bond to the ultrahigh mechanical stability: The ester bond locates at the force concentration point to lock the protein structure to a partially unfolded state with most of the native contacts preserved. On the other hand, the protein structure prevents the ester bond from water attack and makes this chemically labile bond mechanically stable. This stabilization mechanism was verified using molecular dynamics simulation. Moreover, by using disulfide bond mutants, we showed that the unique correlation of the protein structure and the location of the intramolecular bond is responsible to the ultrahigh mechanical stability of the protein domain, regardless what type of bonds at the force concentration point. This can also explain that the ester bond containing proteins show similar mechanical properties as the isopeptide bond containing proteins with similar structures.

Previous bioinformatics analysis suggested that this type of ester bonds is conserved in many cell surface proteins of Gram-positive bacteria. Moreover, these ester bond containing proteins share similar structures despite that they show very low sequence homology^16^. We hypothesized that these proteins also exhibit high mechanical stabilities. To confirm this, we studied the mechanical unfolding of another ester bond containing protein ParV from the Gram-positive bacterium, *Parvimonas sp*. (Supplementary Fig. 19a, b). Similar to the C1 domain of Cpe0147, we did not observe any unfolding signatures of wide type ParV up to a stretching force of 2 nN (Supplementary Fig. 19c). Once the ester bond was deleted by mutation, the protein unfolded at forces of ~200 pN (Supplementary Fig. 19d, e). Therefore, we propose that the chemically labile ester bonds play similar roles as isopeptide bonds to stabilize the structure of Gram-positive surface proteins. In contrast, the structures and the position of the intramolecular bonds of the surface proteins containing thioester are distinct from the proteins containing ester or isopeptide bonds. They are evolved for surface anchoring instead of stabilizing the protein structures^12^.

Why has Cpe0147 evolved such exceptional mechanical stabilities? As one of the major putative MSCRAMMs of *C. perfringens*, it anchors to the cell wall through its C-terminus and projects its N-terminal adhesion domain through 11 repeat ester bond containing Ig domains. The internal ester bonds are strategically positioned to covalently link the first and last β-strands of these Ig domains^9^. Therefore, the stress on the proteins is only propagated from the first β-strand, through the ester bond, and to the final β-strand (β7), leaving the rest of the protein bypassed by the mechanical forces. Mechanical unfolding of these domains may affect the self-assembly and function of these domains or even make them susceptible to proteolytic attack. Their high mechanical stability allows them to maintain folded structures even under mechanical forces of more than 2 nN, which is critical to their biological functions.

**Fig. 7:**
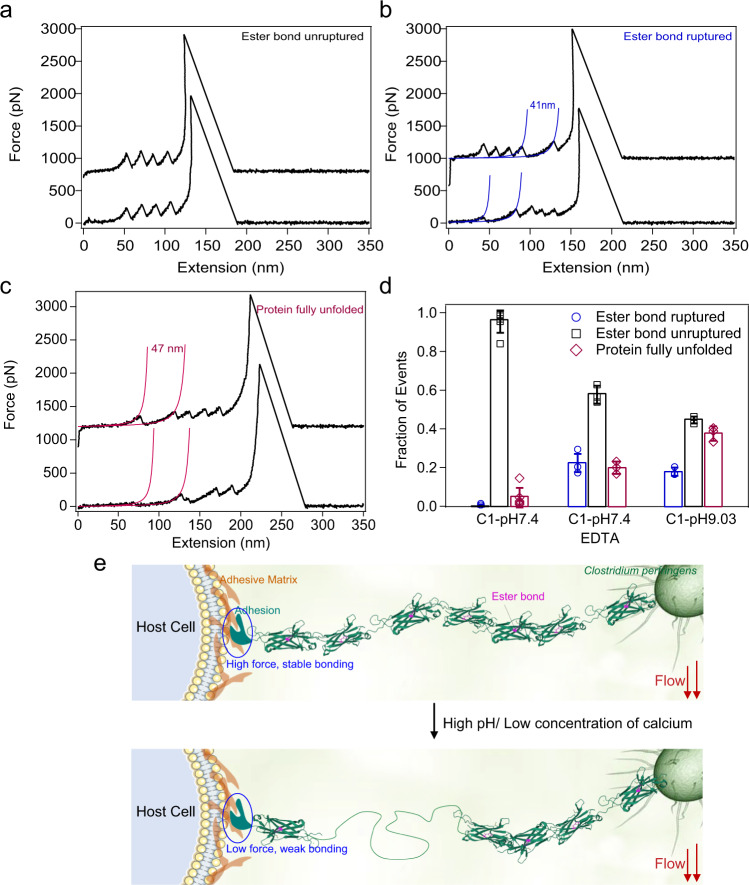
Mechanical features of C1 at a basic pH of 9.0 and in the presence of EDTA (10 mM). **a** Representative force–extension curves of the “Ester bond unruptured” group. The traces show only four GB1 unfolding events without the signature of C1 unfolding or ester bond rupture. **b** Representative force–extension curves of the “Ester bond ruptured” group. Two populations of unfolding events were observed: Four peaks with a Δ*L*_*c*_ of 41 nm relate to GB1 unfolding and an additional one with a Δ*L*_*c*_ of 41 nm corresponds to the rupture of the ester bond. **c** Representative force–extension curves of the “Protein fully unfolded” group. Except for the four GB1 unfolding events, there is an additional peak with Δ*L*_*c*_ of 47 nm (colored in red, upper trace) or a spacer corresponding to the unfolded of C1 (lower trace). **d** The statistics of the three types of events of C1 at pH 7.4 and pH 9.0 and in the presence of EDTA (pH 7.4). The numbers of tests in three conditions are 3, 3, 3 for pH 7.4, pH 7.4 with EDTA and pH 9, respectively. Error bars represent SD. **e** Proposed mechanism for the function of ester bond containing proteins during bacteria invasion. This experiment was repeated three times independently with similar results. Source data are provided as a Source Data file.

Besides strong binding, bacteria also need to develop a mechanism to release the mechanical load to migrate and spread over tissue surfaces for colonization. Especially on wound tissues, the infected bacteria can proliferate quickly and cause inflammation. The wound sites typically show basic pH and low salt concentrations^54^. Previous biochemical studies indicate that the basic pH destabilizes the C1 domain and promotes the hydrolysis of ester bonds^25^. Moreover, the C1 contains two calcium binding sites^16^. Although these calcium binding sites do not contribute to the thermodynamic stability or the ester bond formation^23^, they can change the protein local structural flexibilities. We therefore studied whether the mechanical stability of C1_*WT*_ is affected by some unusual pathological conditions. We pulled the Fgβ-(GB1)_2_-C1_*WT*_-(GB1)_2_-cys following the same experimental scheme as that shown in Fig. 1 under a basic pH of 9.0 or in the presence of a calcium chelation ligand, ethylenediaminetetraacetic acid (EDTA) to remove the calcium ions. Majorities of the force–extension curves were still the same as that under the normal conditions without showing any C1 domain unfolding or ester bond hydrolysis features (Fig. 7a). However, we observed that ~17.9% events at pH 9.0 and ~22.3% events in the presence of EDTA showed clear ester bond rupture events with a contour length increment of ~41 nm (Supplementary Fig. 20a), indicating that the structure of C1 becomes flexible under these conditions (Fig. 7b). As such, they cannot fully protect the ester bond from water attack. Moreover, we observed ~37.5% events at pH 9.0 and ~20.0% events in the presence of EDTA showed the unfolding of the full length C1 domain without the intramolecular ester bond (Fig. 7c). These events either showed a peak with a contour length increment of ~47 nm (Fig. 7c, upper trace, Supplementary Fig. 20b) or showed a long spacer before the first GB1 unfolding peak because the C1 domain was already unfolded before being stretched in the polyprotein (Fig. 7c, lower trace). For comparison, the probability of the ester bond rupture and the C1 domain unfolding events under pH 7.4, pH 9.0, and in the presence of EDTA are summarized in Fig. 7d and Supplementary Tables 1 and 3. Clearly, the pathological conditions can destabilize the C1 domain. It is worth mentioning that in the pathological conditions of slightly basic pH and low calcium concentrations, the C1 domain may still be fully folded, which is different from the experimental conditions in which the pH was set as 9.0 and the calcium ions were almost completely removed by the addition of EDTA.

We propose that destabilizing the stalk region of Cpe0147 may be beneficial for the colonization of the bacteria (Fig. 7e). The N-terminal tip adhesion of cpe0147 contains a thioester bond which can reversibly bind to fibronectin through force regulation. In recent work by Alonso-Caballero et al., they showed that the reactivity of a structural analog of this domain for surface binding is modulated by force-dependent protein folding/unfolding^24^. It undergoes reversible binding and unbinding to surface ligands under forces < 6 pN and remains covalent attachment under higher mechanical stress. Unlike the tip adhesin, the stability of the stalk domains is critical for the force propagation to the tip adhesin. When the Ig-fold of the stalk domains is folded, they can efficiently transduce forces to the tip adhesin for strong surface anchoring. When they are partially unfolded, they can release the force on tip adhesin and promote its detachment from the tissue surfaces for migration. Therefore, the type and the position of the intramolecular bonds in the tip adhesin and the stalk domains are distinct. However, they are both evolved for efficient host surface binding, biofilm formation, and immune evasion. Further experimental work is needed to validate this hypothesis.

It remains unknown whether Cpe0147 can bind to the host or other cell adhesion proteins via the ester bond containing stalk region^55^. If so, we hypothesize that the force direction can be altered by such binding events and eventually cause the local unfolding of the protein structure and expose the ester bond to water, similar to the circular permutant of C1 studied in this work. This can also lead to the hydrolysis of the ester bond, then complete unfolding of these domains, and eventually loss of adhesion. In contrast, the isopeptide bond containing proteins can only partially unfold in this scenario and refold back after the release of force due to the high stability of isopeptide bonds.

In summary, we employed AFM-based single-molecule force microscopy, protein engineering, and molecular dynamics simulation to study the mechanical properties of an ester bond containing protein, the C1 domain of Cpe0147 from Gram-positive bacterium, *C. perfringens*. We find that despite that individual ester bond is mechanically much weaker than an isopeptide bond, C1 exhibits similar mechanical stability as the isopeptide bond containing protein and does not completely unfold even under forces > 2 nN. The ester bond locks the structure to a partially unfolded conformation, in which the ester bond remains largely water inaccessible. This allows the ester bond to withstand considerable mechanical forces and in turn prevent complete protein unfolding. Breaking this structural correlation by circular permutation leads to sequential protein unfolding and ester bond hydrolysis. Destabilizing the protein structure at basic pHs or low calcium concentrations also reduces the protecting effect and leads to the hydrolysis of the ester bond. We propose that the environment-dependent mechanical response of Cpe0147 may allow the bacteria to smartly switch between binding and unbinding states for better invasion. We further highlight the importance of the position of ester bond on the high mechanical strength of the protein by engineering a mutant with a disulfide bond at the ester bond location that can retain high mechanical stability in the presence of reducing agents. These results provide the molecular mechanism on the high mechanical stability of ester bonding containing protein and might also inspire the design of new antibiotics by mechanically destabilizing these proteins.

## Methods

### Chemicals

All chemicals used were supplied by Sigma-Aldrich (USA) or New England Biolabs (USA) if not specified explicitly.

### Protein engineering

The genes for C1 domain of Cpe0147 from *C. perfringens*, Fgβ/SdrG N2N3 domain from *Staphylococcus epidermidis*, and ParV from *Parvimonas sp*. were custom synthesized codon-optimized for expression in *Escherichia Coli* (GenScript, China), and designed to include 5′ *Bam*HI, 3′ *Bgl*II, and *Kpn*I restriction sites. The genes encoding the chimeras used in this work were constructed in the pQE80L vector (Qiagen, Germany) or pET22b vector (Novagen, USA) based on the same sticky end generated by *Bam*HI and *Bgl*II following standard molecular biology techniques^56^. Point mutations and circular permutations were created through polymerase chain reactions. The constructed plasmids were sequenced to confirm the correct protein sequence. All these constructs and full amino acid sequences are shown in Supplementary Information.

### Protein expression and purification

All proteins used in this work were expressed in *E. coli* (BL 21) cells and purified with Co^2+^ affinity chromatography using TALON resins (Takara, USA). Purified protein samples were dialyzed to remove the imidazole that used for protein elusion and stored in PBS buffer at 4 °C until use. Protein concentrations were measured by spectrophotometry at 280 nm with typical final concentrations of ~100 μM (Thermo NanoDrop 2000, USA).

### AFM sample preparation

More detailed AFM-based single-molecule force spectroscopy protocol has been published previously^28,39^. In brief, AFM cantilevers (Bruker, MLCT, USA) and glass surfaces (Sail Brand, China) were modified with aminosilane.

Glass surface: Glass substrates were cut into 1 × 1 cm^2^ slides, soaked in a freshly prepared chromic mixture overnight, thoroughly washed with deionized (DI) water, ethanol, and acetone successively, and then dried under a steam of nitrogen to produce surfaces with exposed hydroxyl groups. These substrates were immersed in an anhydrous toluene solution containing 1% (v/v) APTES (Merck, USA) at room temperature (R.T.) for 1 h for amination. Then, they were washed with toluene and ethanol, dried under a nitrogen flow. Finally, surfaces were incubated at 90 °C for 30 min. Glass substrates were stored in a desiccator under argon and typically used within half a month.

Cantilevers: Silicon nitride (Si_3_N_4_) cantilevers (MLCT-D, Bruker) were first cleaned with Milli-Q water, and then placed in a chromic mixture (chromic acid) at 80 °C for 30 min. After that, the cantilevers were washed with DI water, then ethanol, and dried under a steam of nitrogen. Then, the hydroxylated cantilevers were immersed in an anhydrous toluene solution containing 1% (v/v) APTES for 1 h. After that, they were rinsed with toluene, then ethanol, dried under a nitrogen stream, and incubated at 80 °C for 45 min. Finally, they were stored overnight under argon and used in the following steps.

Both glass substrates and cantilevers were immersed in DMSO containing 0.2 mM Mal-PEG-NHS (MW: 5000 Da, Nanocs, USA) for 1 h. After being washed with DMSO, ethanol, and dried under a nitrogen stream, the resulting maleimide-coated glass substrates and cantilevers were kept dry at −20 °C before use in the following protein modification steps in the single-molecule experiments.

### AFM-based single-molecule force spectroscopy

The force spectroscopy experiments were carried out using a commercial JPK ForceRobot 300 AFM system (JPK Instruments AG, Germany). Experiments were conducted at R.T. (22 °C) and performed in 10 mM PBS buffer with or without 10 mM TCEP if needed. Soft silicon nitride MLCT-D cantilevers of typical spring constant of 30–45 pN nm^−1^ were used for all experiments and calibrated using the thermal tune method using the data acquisition software from JPK after allowing the cantilever to equilibrate in solution for at least 30 min. To transform the electrical signals from the photodiode to the actual displacement, the optical lever sensitivity and its inverse was determined by pushing the cantilever tip to the glass substrate and assumed the bending of the cantilever equal to the movement of the piezoelectric positioner. We did not correct the bending angles of the cantilever, which may lead to ~10% errors to the estimated spring constants^57^. In a typical pulling experiment, cantilevers were briefly and gently (~300 pN) brought in contact with the functionalized surface and held at the surface for 0.5 s, then retracted at a constant velocity of 1.6 μm s^−1^. The sampling frequency was 10 kHz and the records were smoothed by the moving average of 10 points. The force–extension curves were recorded using JPK data processing software and were further analyzed by a custom-written procedure in Igor 6.37 (Wavemetric Inc).

We only used the curves containing four peaks of contour length increments of ~18 nm corresponding to the unfolding of the GB1 domains for data analysis. The contour length increment was determined by fitting the force peaks using the worm-like chain model with persistence lengths in a range of ~0.2–0.5 nm. The noise level of the force–extension curves was ~10 pN. Only the peaks with rupture forces higher than three times the noise level were included in the force histogram. The contour length increment was determined by fitting the consecutive peaks using the WLC model with fitting residuals < 20 pN. In the cases that the traces only show a spacer instead of a detectable peak, we assigned them as the signature of the unfolded protein domain, because the unfolding of the protein structure is the prerequisite for the hydrolysis of the ester bond.

### Molecular dynamics simulations

The molecular dynamics simulations were conducted by the GROMACS 2019 software^58^ with the ff14SB force field^59^ and TIP3P water^60^. To model the ester bond, we introduced a covalent bond between the Thr-11 (Oγ1) and Gln-141 (Cδ). The extra atoms (Hγ1 of Thr-11; Nε2, Hε21, and Hε22 of Gln-141) were removed, with their partial charges being integrated into the nearby heavy atoms. The atomic coordinates of the C1_*WT*_ were taken from the Protein Data Bank (entry 4MKM)^16^. The five N-terminal residues lacking structural information were not included. In constructing the C1_*CP*_, a ELP linker with the length of 20 amino acids was added between the two termini of the C1, and the residues Leu-125 and Asp-126 were used as the new termini. The three-dimensional structure of the linker was modeled by the ModLoop^61,62^.

The C1_*WT*_ and C1_*CP*_ were solvated in the rectangular water boxes with the dimensions of ~241 Å × 84 Å × 85 Å (with 48891 water molecules) and 1017 Å × 74 Å × 74 Å (with 159145 water molecules), respectively. Sodium ions were added to neutralize the systems. The LINCS algorithm was used to restrain the covalent bond involving hydrogen atoms^63^. After a 50,000-step minimization using the steepest descent method, each system was equilibrated for 0.1 ns in the NVT ensemble and another 0.1 ns in the NPT ensemble. The temperature and pressure were controlled at 298.0 K and 1.0 atm, respectively. The heavy atoms of the proteins were restrained to their original positions by a harmonic potential during the minimization and equilibrium steps. Starting from the equilibrated structures, we performed steered MD simulations by applying pulling force between the termini residues along the x-axis in NVT ensemble. For the C1_*WT*_, the constant pulling force were applied to the termini residues with the strength of 0 and 1500 pN and the simulations lasted for 50 ns. For the C1_*CP*_, we firstly conducted a constant velocity pulling simulations with the pulling speed of 49 Å/ns, such that the ester bond stars to sustain pulling force. Then, we performed the same constant pulling force simulations as that in the C1_*WT*_. Eight independent simulations were conducted for each system. To increase the statistics, we also performed another two (one) constant pulling force simulations with the length of 35 ns for the C1_*WT*_ (C1_*CP*_). The snapshots sampled during the constant pulling force simulations were used for analysis with the snapshots corresponding to the first 5 ns being omitted. The softwares MDTraj^64^ and PyMOL were used for the structure visualization.

### Reporting summary

Further information on research design is available in the Nature Research Reporting Summary linked to this article.

## Supplementary information

Supplementary Information

Reporting summary

## Data Availability

The source data underlying all figures are provided as Source Data file. All other data that support the findings of this study are available for download from https://biomech.nju.edu.cn/Download/index.html. Source Data are provided with this paper.

## Source data

Source Data

## Code availability

The AFM code that supports the findings of this study is available from https://biomech.nju.edu.cn/Download/index.html.
