# Supplementary material for: An ester bond underlies the mechanical strength of a pathogen surface protein
Source: Nat Commun. 2021 Aug 23;12:5082. doi: 10.1038/s41467-021-25425-6 (PMC8382745; doi:10.1038/s41467-021-25425-6)
Supplement: Supplementary file 1 — Supplementary Information [file 41467_2021_25425_MOESM1_ESM.pdf]

Supplementary Information

An ester bond underlies the mechanical strength of a pathogen surface protein

Lei et al.

Supplementary Information

An ester bond underlies the mechanical strength of a pathogen surface protein

Hai Lei[1,2#], Quan Ma[1#], Wenfei Li[1#], Jing Wen[3], Haibo Ma[3], Meng Qin[1], Wei Wang[1] and Yi Cao[1,2]*

[1]Collaborative Innovation Center of Advanced Microstructures, National Laboratory of Solid State Microstructure, Department of Physics, Nanjing University, Nanjing, China 210093

[2]Chemistry and Biomedicine innovation center, Nanjing University, Nanjing, China 210093

[3]Key Laboratory of Mesoscopic Chemistry of MOE, School of Chemistry and Chemical Engineering, Institute of Theoretical and Computational Chemistry, Nanjing University, Nanjing, China 210023

[#]equal contribution.

*E-mail: caoyi@nju.edu.cn

Supplementary Notes

Supplementary Note 1:

The maleimide-thiol chemistry has been widely used for single molecule force spectroscopy experiments. In most cases, the systems of interest ruptures at much lower forces than the detaching forces of the maleimide-thiol adducts (< 1 nN). It is safe to assume that the maleimide-thiol bond is not mechanically labile. In the remarkable work on the mechanical stability of SdrG:Fgβ complex by Milles L.F. et al.[1], they reported the rupture forces of ~ 2 nN. In our previous work by pulling the succinimide formed between the surface linked thiol and a maleimide at the end of a PEG linker, we found that the rupture forces were ~1 nN. It seems that the maleimide-thiol bond may have distinct mechanical stabilities on various surfaces or proteins. In our own laboratory, we also found that the detaching forces for covalently attached proteins to the substrate using the maleimide-thiol chemistry can vary dramatically, depending on the location of the thiol groups. A possible reason can be that the local chemical environment of the thiol group can affect the hydrolysis of the succinimide ring. As reported by Shen, B.-Q. et al.[2], the positively charged environment of proteins can promote the hydrolysis of the succinimide ring considerably. Moreover, the chemical environment of the substrates can also affect the rupture forces. In some cases, due to the favorable chemical environment, the maleimide-thiol adducts hydrolyze very fast even in the absence of stretching forces. Therefore, the detaching forces can vary markedly for various systems even though they used the maleimide-thiol conjugation method.

Supplementary Note 2:

In the case of the SdrG:Fgβ tethering on the cantilever tip, the formation and dissociation of the SdrG:Fgβ complex are reversible. The maleimide-thiol bond experiences stretching forces every time when a polyprotein is picked up and pulled. This can lead to either dissociation of the maleimide-thiol bond or hydrolysis of the succinimide ring to stabilize the bond. After a few trials, all the SdrG proteins that still attach to the cantilever tip are linked through hydrolyzed succimimide ring, which is a strong linker and can survive at stretching forces over 2 nN. Therefore, most of the events showed detaching forces over 2 nN in our experiments. In the case of SpyCatcher/SpyTag chemistry for picking up the polyprotein, because SpyCatcher/Spytag forms strong covalent linkage, the rupture position should be the weakest bond in the whole linkage (most likely the maleimide-thiol bond). Therefore, every SpyCatcher on the cantilever tip can only be used to pick up a polyprotein from the substrate once and then becomes a "dead" tether. The rupture events correspond to the dissociation of freshly formed maleimide-thiol bonds and typically occur at forces of ~1 nN[3].

We do not think the interactions at 1 nN are non-specific. If so, we would observe most events of less than four GB1 domains. In contrast, most of the events (>95%) with detaching forces of ~1 nN show four GB1 unfolding events, indicating that the polyproteins are stretched between the N- and C- termini. Moreover, the pickup rates for the SpyCatcher/SpyTag method gradually decrease with time and typically only ~30-50 events can be obtained for each cantilever. If the polyproteins are picked up by non-specific interactions, we would expect that the pickup rates remain constant, and more events can be obtained in each experiment.

It is worth mentioning that there is also the possibility that some events were picked up by high non-specific adhesion forces instead of specific interactions. However, these events did not affect

the conclusion of this paper in a significant way.

Supplementary Note 3:

According to the Instructions from Thermo Scientific (Cat# 20490, 20491), "Experiments indicate that TCEP completely oxidizes within 72 hours in 0.35 M phosphate-buffered saline (PBS), pH 7.0. Approximately 50% oxidation occurs in the same amount of time in 0.15M PBS, pH 8.0." In our experiments in 10 mM PBS at pH 7.4, it is unlikely TCEP is degraded during the experiments. We did not see the difference in the rupture forces in the data from the first four hours and the data from the second four hours.

Supplementary Note 4:

Note that the ΔLc of the "Disulfide ruptured" group is ~41 nm, which is shorter than the ΔLc of the "Disulfide unformed" group (~47 nm). This suggests that while the C1-df with the disulfide bond can withstand significant stretching forces, it is partially unfolded to the disulfide bond position at high forces with a ~6 nm shortening of the contour length. However, in these events, we did not observe the transition from the folded state to the partially unfolded state in the force-extension curves, which may indicate that C1-df is already at the partially unfolded state prior to the measurement (Supplementary Figure 17). Introducing the disulfide bond may reduce the thermodynamic stability of the protein, making the protein occasionally adopt the unfolded conformation even the disulfide bond is formed in the protein structure. In contrast, wild type C1 cannot spontaneously unfold to make the ester bond solvent accessible due to its high thermodynamic stability.

Supplementary Figures

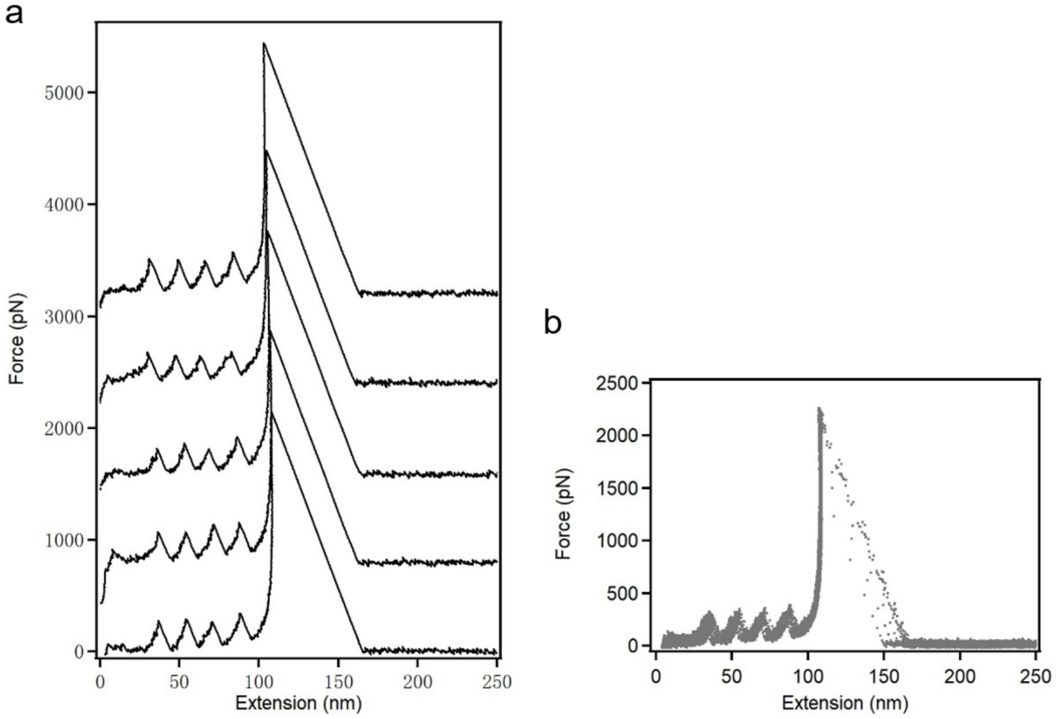

Supplementary Figure 1. Single molecule force spectroscopy experiments of the polyprotein Fgβ-(GB1)$_2$-C1$_{WT}$-(GB1)$_2$-cys. **a**, Five represent force-extension curves. **b**, Overlay of twenty force-extension curves. There are only four peaks caused by the unfolding of GB1 and the last one due to the rupture of Fgβ/SdrG complexes.

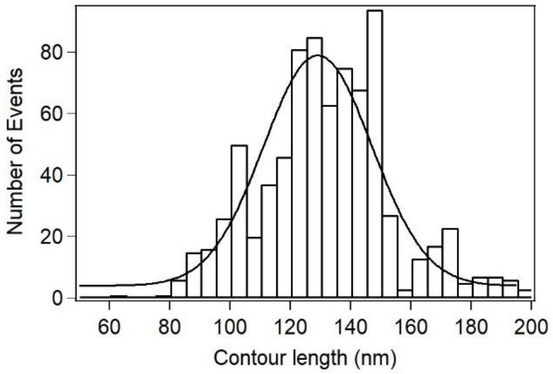

Supplementary Figure 2. The distribution of the contour length of the last peak in the single-molecule force-extension traces in Fig. 1f.

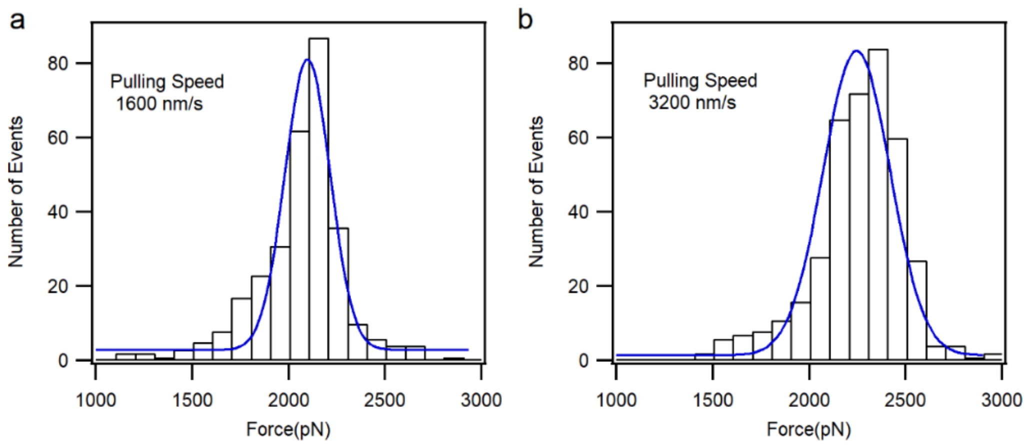

Supplementary Figure 3. Rupture force distributions of Fgβ/SdrG in the single-molecule force spectroscopy experiments at the pulling speed of 1600 nm/s (a) and 3200 nm/s (b), respectively.

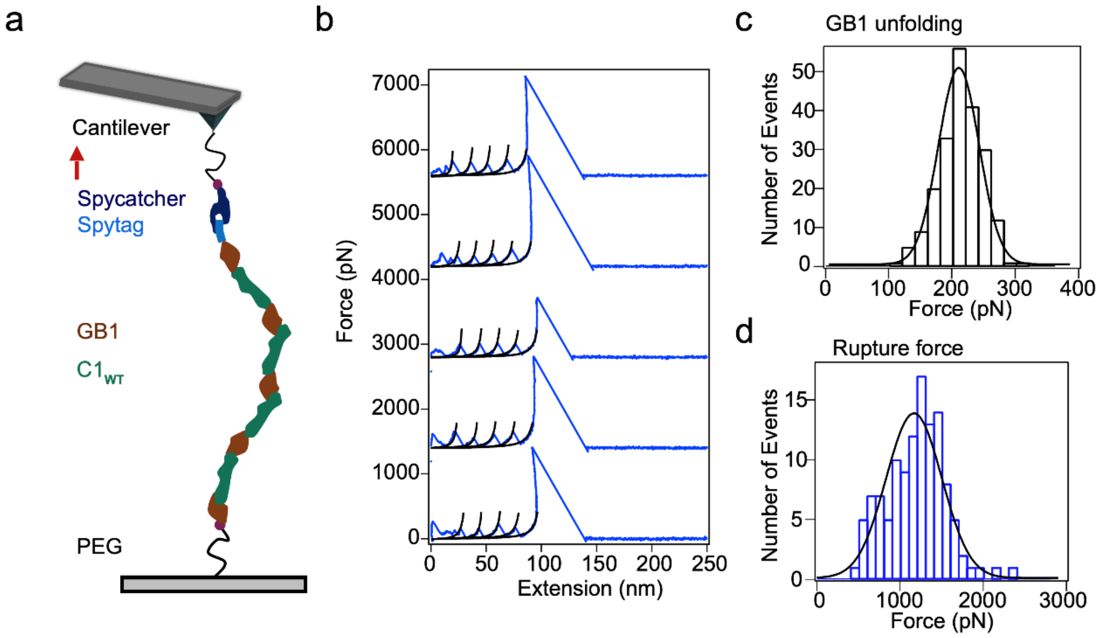

Supplementary Figure 4. Single molecule force spectroscopy experiments on the C1$_{WT}$ containing polyprotein, Spytag-(GB1-C1$_{WT}$)$_4$-cys, using the Sypcatcher/Spytag chemistry. **a,** Schematic of the single molecular force spectroscopy experiment. **b**, Typical force-distance traces. Worm-like chain (black) was used to fit the saw-tooth peaks. The last peak may be due to the rupture of the maleimide-thiol adduct for surface covalent linking. **c**, Unfolding force distribution of the fingerprint maker GB1 proteins. Gaussian fitting shows the average unfolding force of 205.68 ± 45.47 pN. (mean ± S.D.) (the total number of unfolding events, n=212). **d**, The detaching forces of the polyprotein either from the substrate or the cantilever shows an average force of 1167.9 ± 473.5 pN. (mean ± S.D.) (the total number of events, n=119).

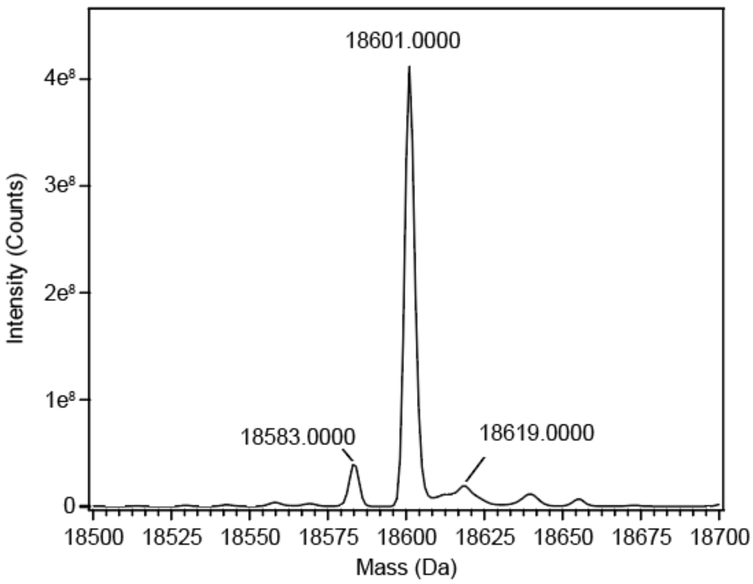

Supplementary Figure 5. Mass spectroscopy of the wide type of C1 protein. The ester bond-formed (18601 Da calculated and observed) protein is dominant and only little population is ester bond-unformed protein (18619 Da calculated and observed).

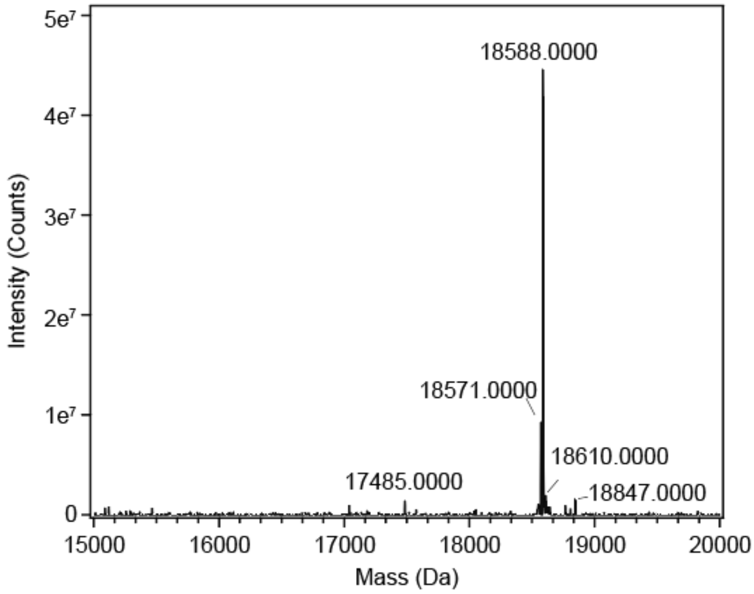

Supplementary Figure 6. Mass spectroscopy of the mutant of C1 protein ($C1_{T11A}$). The mass calculated (18587 Da, ester bond unformed) and observed from the mass spectroscopy (18588 Da) are consistent.

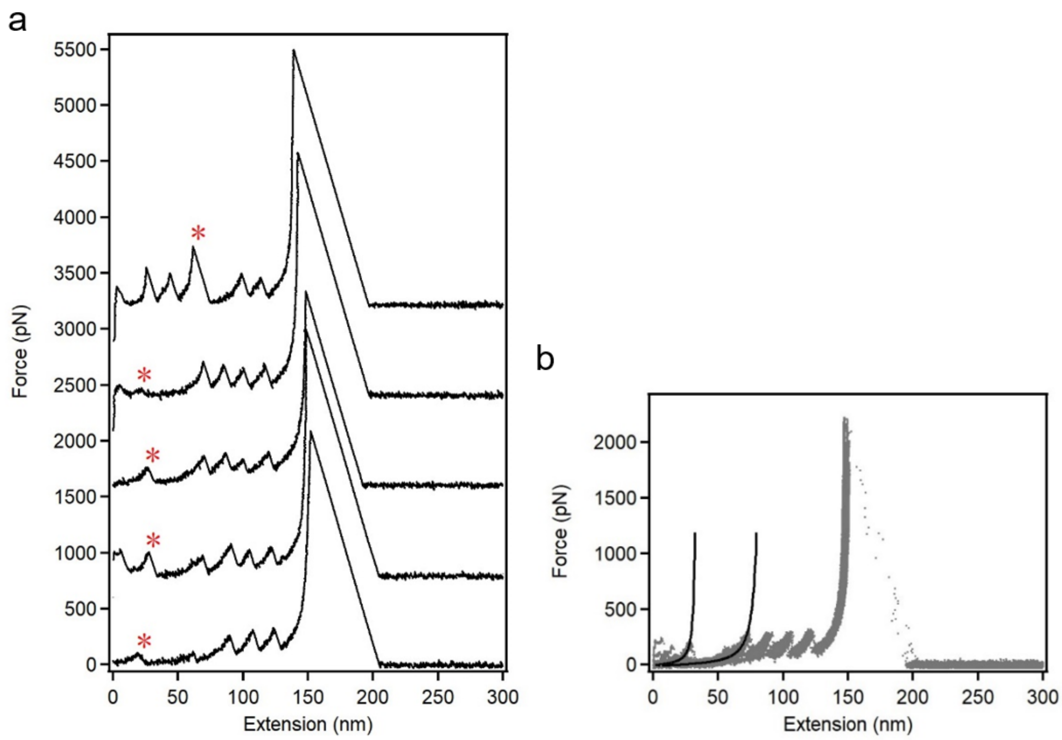

Supplementary Figure 7. Single molecule force spectroscopy experiments of the polyprotein Fgβ-(GB1)$_2$-C1$_{T11A}$-(GB1)$_2$-cys. **a**, Five represent force-extension curves. The peaks marked with "*" correspond to the unfolding of C1$_{T11A}$. **b**, Overlay of twenty force-extension curves. There are six peaks in the curves. With WLC fitting, the peak with a Δ*Lc* of 47 nm corresponds to the unfolding of C1$_{T11A}$, the next four peaks relate to the unfolding of GB1 and the last one due to the rupture of Fgβ/SdrG complexes.

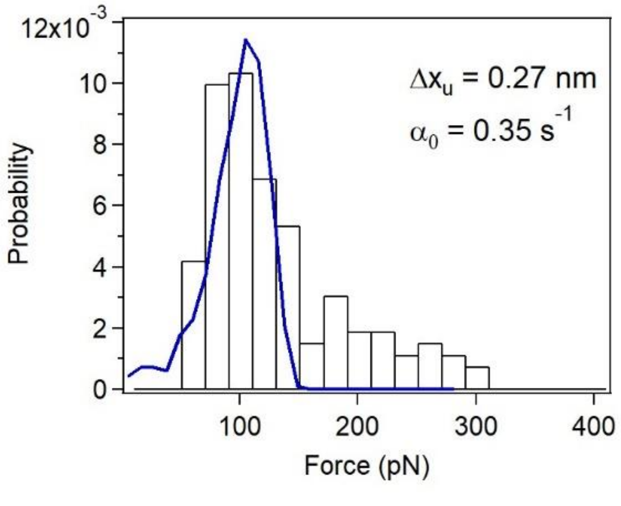

Supplementary Figure 8. Monte Carlo simulation for the unfolding force of C1$_{T11A}$ using Bell-Evans model.

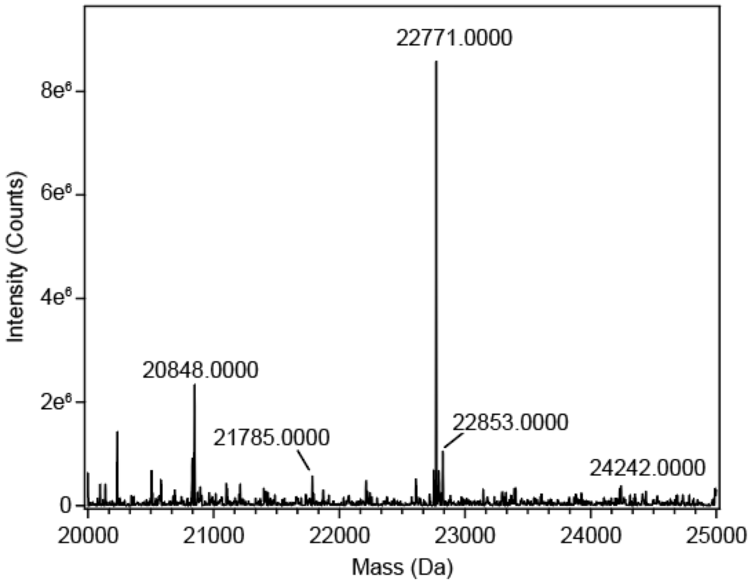

Supplementary Figure 9. Mass spectroscopy of the circular permutant of C1 protein (C1$_{CP}$). The ester bond-formed (22770 Da calculated and 22771 Da observed) protein is dominant.

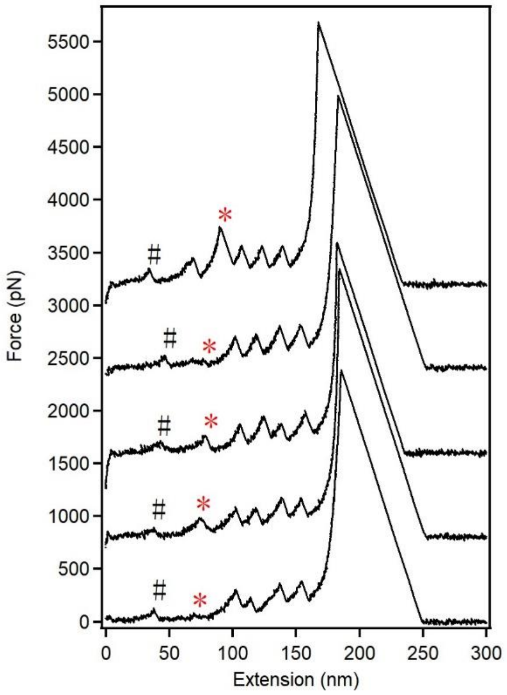

Supplementary Figure 10. Five force-extension curves of single molecule force spectroscopy experiments of the polyprotein Fgβ-(GB1)$_2$-C1$_{CP}$-(GB1)$_2$-cys.

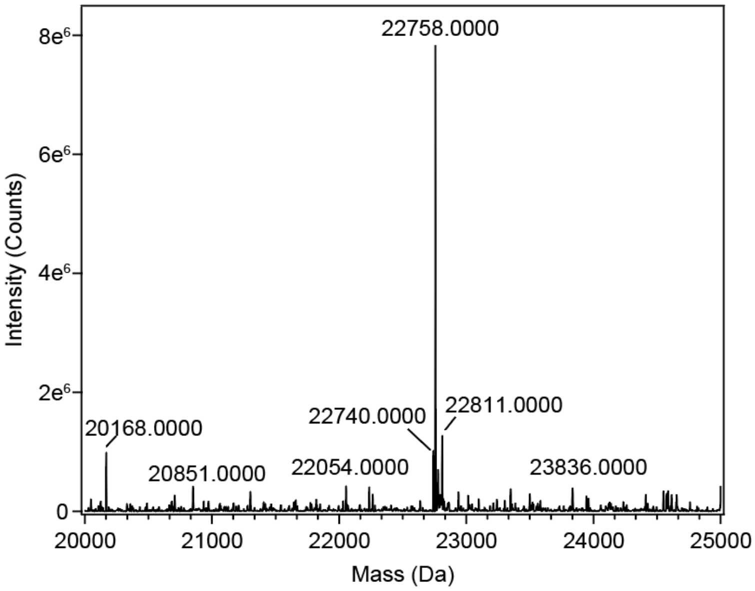

Supplementary Figure 11. Mass spectroscopy of the circular permutant of C1$_{CP}$ protein (C1$_{CP}$-T11A) without ester bond. The calculation mass is 22758 Da which coordinates to the experiment data (22758 Da).

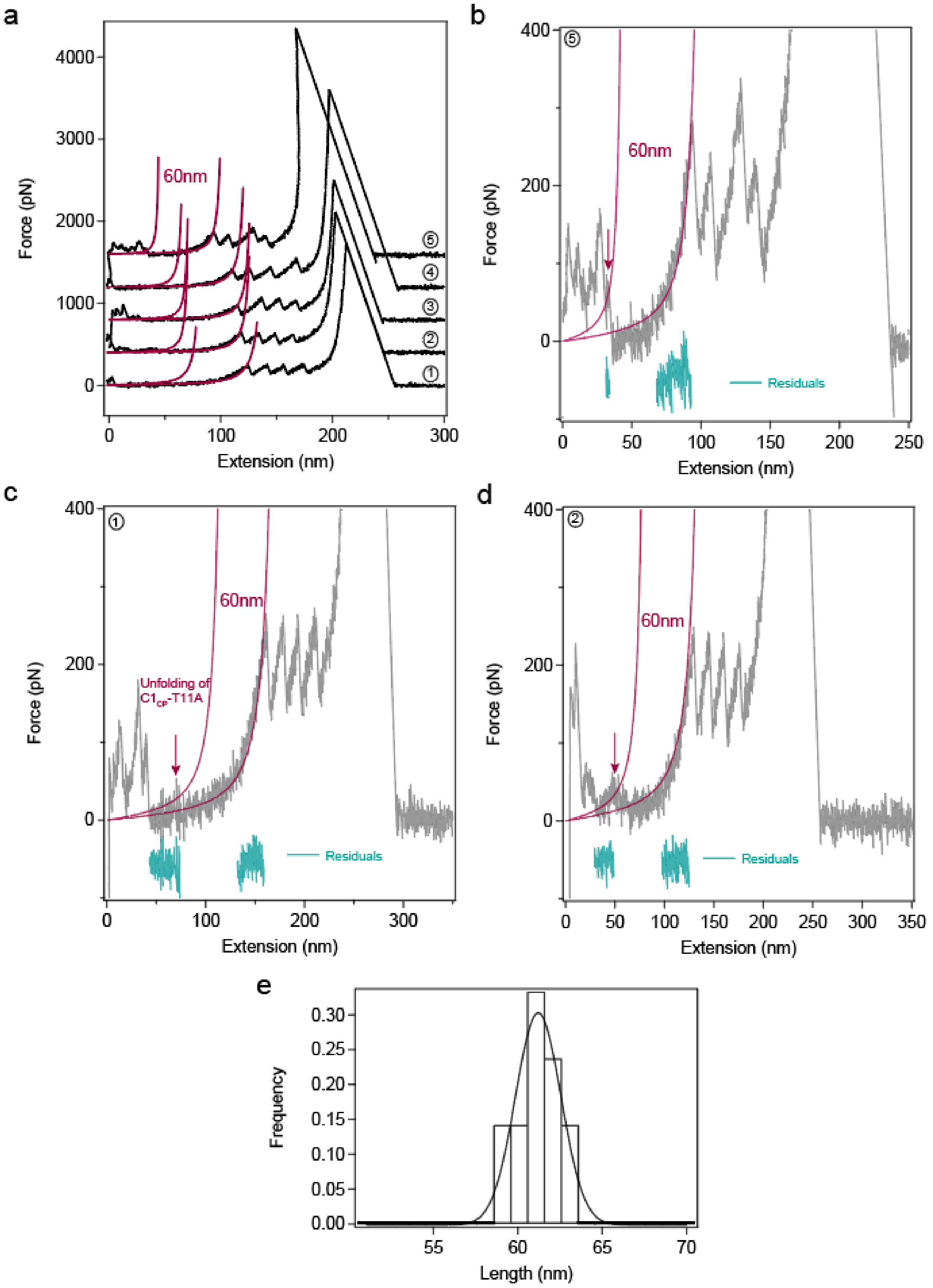

Supplementary Figure 12. a, Five force-extension curves of single molecule force spectroscopy experiments of the polyprotein Fgβ-(GB1)$_2$-C1$_{CP}$-T11A-(GB1)$_2$-cys. **b**, **c** and **d**, Zoom in of curve 1, 2 and 5 in **a**. **e**, Histogram of the contour length increment for the unfolding of C1$_{CP}$. The average is 60.7 ± 1.9 nm by Gaussian fit (the total number of events, n=21).

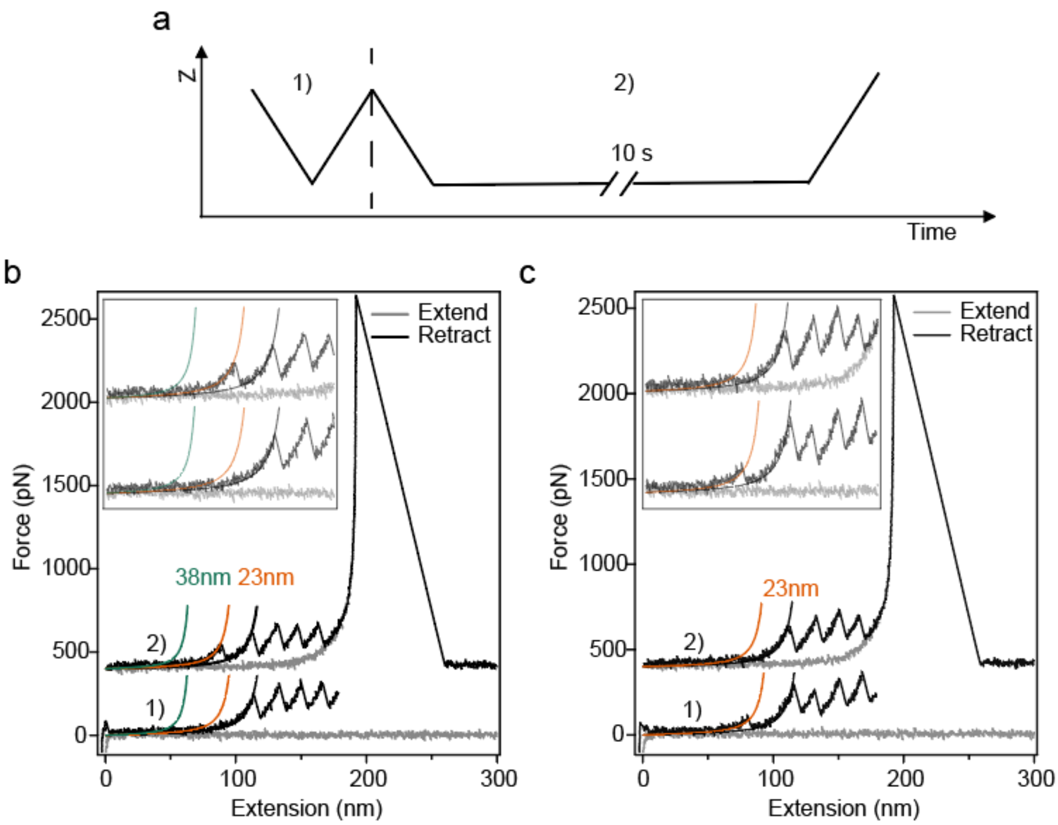

Supplementary Figure 13. Reversible unfolding and refolding of $C1_{CP}$ observed directly during repeated stretching and relaxation experiments. **a,** Scheme of the refolding experiments. In the first cycle, the AFM tip approaches the substrate with a constant pulling speed and is retracted to a proper position to fully unfold the polyprotein and avoid detaching it. In the second cycle, the tip re-extends to the substrate surface and holds for 10 s to allow the protein domains to refold. Then the tip is full retracted to break the molecule. The refolding ratio is 11.07% (28/253) in ~10 s. Two successful refolding examples are shown in **b** and **c**. The peaks with the green fitting curves display a ΔL_c of ~38 nm, corresponding to the mechanical unfolding of $C1_{CP}$. The peaks with the orange fitting curves display a ΔL_c of 23 nm, corresponding to the rupture of the intramolecular ester bond. Note that, due to detection limit of our AFM setup (~30 pN), the unfolding forces of $C1_{CP}$ may not be detectable in some traces.

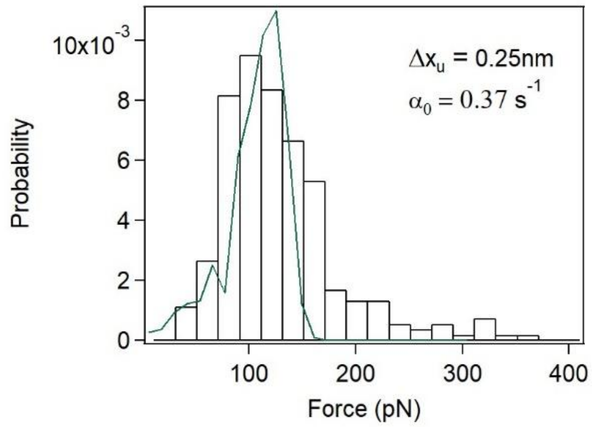

Supplementary Figure 14. Monte Carlo simulation for the unfolding force of C1$_{CP}$ using the Bell-Evans model.

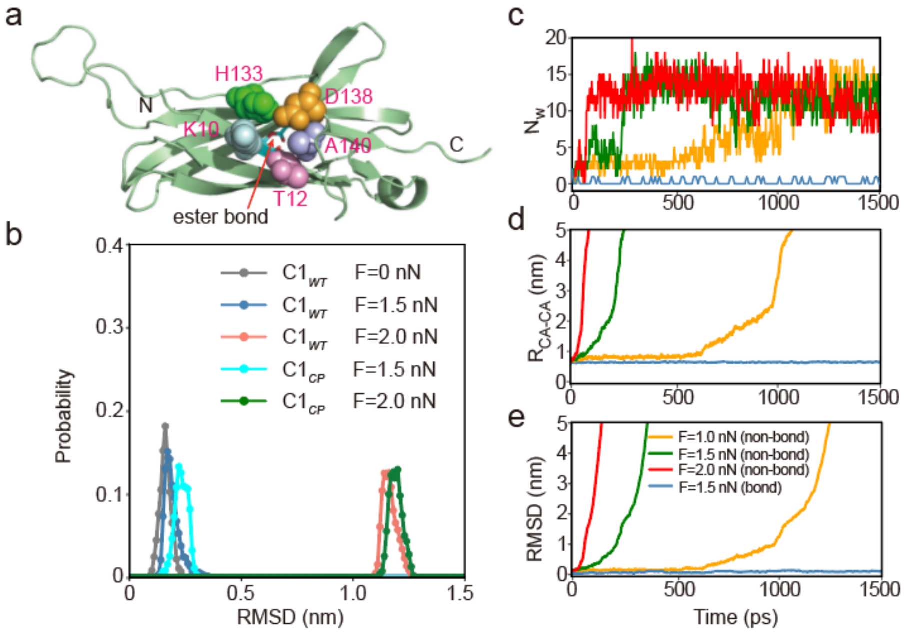

Supplementary Figure 15. Mutual interplay between the ester bond and the local structure of its surrounding residues under pulling forces. **a**, Cartoon representation of the native structure of the C1$_{WT}$. The residues immediately surrounding the ester bond (including K10, T12, H133, D138, and A140) were shown by sphere representation. These residues form a barrel-like structure preventing the water molecules from attacking the ester bond. The two residues forming the ester bond (T11 and N141) were shown by stick representation. **b**, Distributions of the rmsd of the local structure surrounding the ester bond (involving the residues K10, T12, H133, D138, A140, T11, and N141) compared to that in the native structure for the C1$_{WT}$ and C1$_{CP}$ under different pulling forces. **c**, Representative MD trajectories showing the number of accessible water molecules to the ester bond (N_W) as a function of time for the simulations with the covalent-bonding interactions arising from the ester bond deleted (non-bond) at different pulling forces. For comparison, the result with the ester bond formed (bond) at the pulling force of 1.5 nN was also shown. **d-e**, Same as (**c**) but showing the distance between the two C_α atoms of the ester bond forming residues (**d**) and the rmsd of the local structure surrounding the ester bond (**e**) as a function of time.

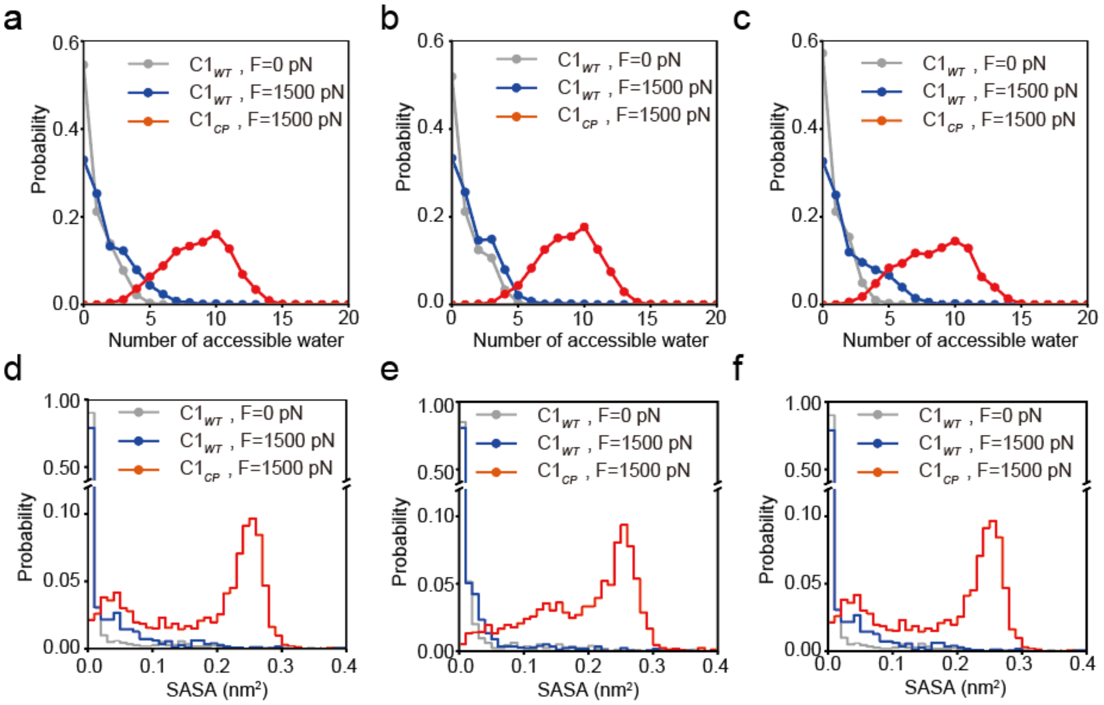

Supplementary Figure 16. Molecular dynamics simulation (**a-c**) Distributions of the number of water molecules within 5Å from the Oε1 atom of the Gln141 for C1$_{WT}$ (blue) and C1$_{CP}$ (red) at the constant pulling force of 1500 pN calculated based on the full-length trajectories (**a**), the first half of the trajectories (**b**), and the second half of the trajectories (**c**). The snapshots corresponding to the initial 10 ns were not included. For comparison, the result for C1$_{WT}$ (grey) without applying force was also shown. (**d-f**) Distributions of the solvent accessible surface area (SASA)[4] for the Oε1 atom of the Gln141 for C1$_{WT}$ (blue) and C1$_{CP}$ (red) at the constant pulling force of 1500 pN calculated based on the full-length trajectories (**d**), the first half of the trajectories (**e**), and the second half of the trajectories (**f**). The significant differences of the number of accessible water (and the solvent accessible surface area) between the C1$_{WT}$ and C1$_{CP}$ for the full-length trajectories, the first half of the trajectories, and the second half of the trajectories suggest reasonable convergence of the simulations.

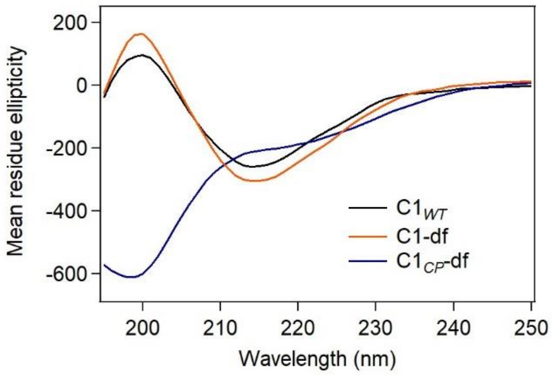

Supplementary Figure 17. CD data for C1$_{WT}$ (black), C1-df (orange) and C1$_{CP}$-df (blue) proteins. The C1$_{WT}$ and C1-df proteins show folded all β-sheet structures with a negative peak at ~215 nm. However, the C1$_{CP}$-df shows shallower peak at ~215 nm and a major negative peak at ~198 nm, corresponding random coil structures. This indicates that the C1$_{CP}$-df structure is flexible or partially unfolded.

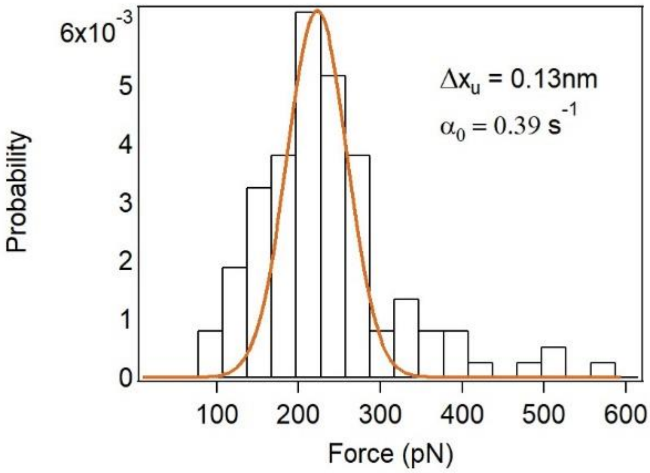

Supplementary Figure 18. Monte Carlo simulation for the rupture force of disulfide bond in C1$_{CP}$-df using Bell-Evans model.

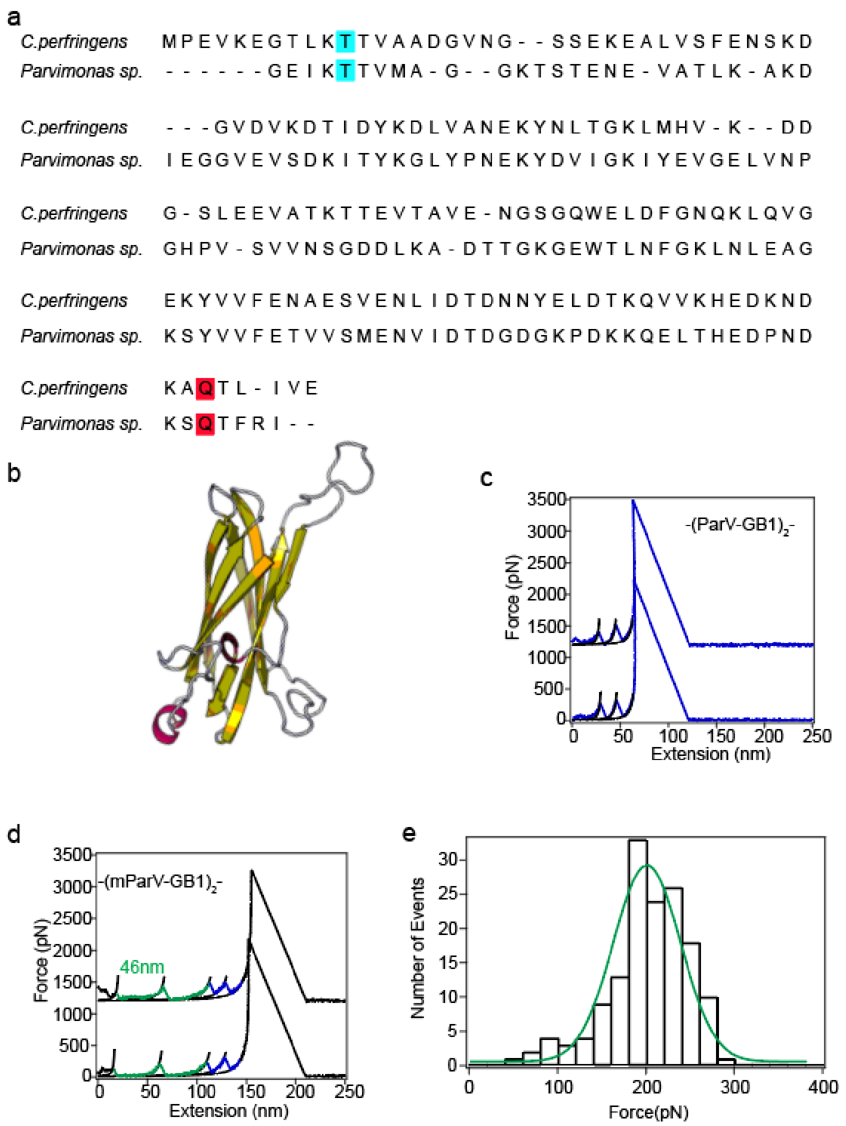

Supplementary Figure 19. Mechanical unfolding of ParV and its ester bond deletion mutant (mParv). **a**, Sequence alignment of C1 from *C. perfringens* and ParV from *Parvinmonas sp.*. In both proteins, the ester forming residues Thr (Cyan) and Gln (Red) are conserved. **b**, Predicted structure of ParV, which is similar to the C1 structure. **c**, Representative force-extension curves of pulling Fgβ-(ParV-GB1)$_2$-cys by a SdrG-cys modified cantilever. Force-extension curves only display two unfolding events which are attributed to the unfolding of GB1. **d**, Representative force-extension curves of pulling Fgβ-(mParV-GB1)$_2$-cys by a SdrG-cys modified cantilever. The two peaks (colored in green) with Δ*Lc* of 46 nm correspond to the unfolding of mParV domains; the next two peaks (colored in blue) correspond to the unfolding of GB1; and the last peak correspond to the rupture of the Fgβ/SdrG complex. **e**, Unfolding force distribution of mParV. The unfolding forces of mParV is higher than that of the C1 domain of Cpe0147 (~100 pN), despite that they share similar structures.

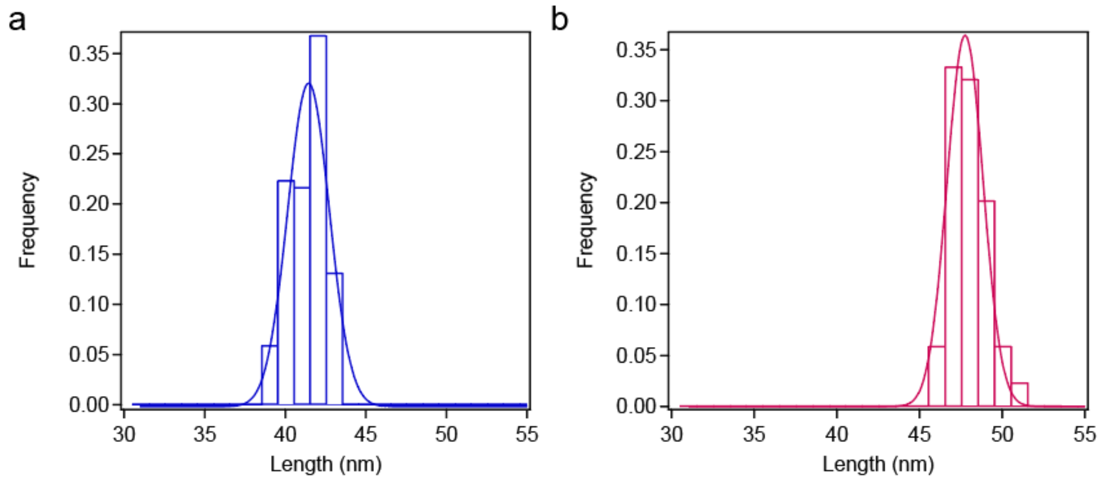

Supplementary Figure 20. The histograms of the contour length increment for the ester bond rupture (a) and protein full unfolding (b). Gaussian fits (solid lines) to the experimental data show that the contour length increment is 40.9 ± 1.8 nm (mean ± S.D.) for the ester bond rupture (total number of rupture events, n=152) and 47.3 ± 1.6 nm (mean ± S.D.) for the protein full unfolding (total number of unfolding events, n=84).

Supplementary Tables

Supplementary Table 1. Summary of the single-molecule experiments for wide type C1 at different pHs.

Experiment	Pulling speed (nm/s)	Total number	Single-molecule number	Single-molecule ratio (%)	Folded number	Fully unfolded number	Ester bond rupture number	Spring constant (pN/nm)
$C1_{WT}$ (pH 7.4)	1600	4110	69	1.67	58	10	1	35.8
	1600	19590	223	1.14	219	4	0	34.7
	1600	14903	157	1.05	152	4	1	35.2
	1600	18027	196	1.09	194	2	0	31.7
	1600	14200	94	0.67	93	1	0	29.1
	1600	11528	66	0.57	63	3	0	38.6
$C1_{WT}$ (pH 9.0)	1600	10216	85	0.83	38	33	14	40.32
	1600	28741	569	1.98	243	230	96	38.8
	1600	28416	531	1.87	246	177	108	42.9

Supplementary Table 2. Summary of the single-molecule experiments for wide type C1 at different pulling speeds.

Experiment	Pulling speed (nm/s)	Total number	Single-molecule number	Single-molecule ratio (%)	Folded number	Fully unfolded number	Ester bond rupture number	Spring constant (pN/nm)
$C1_{WT}$	1600	4110	69	1.67	58	10	1	35.8
	1600	19590	223	1.14	219	4	0	34.7
	1600	14903	157	1.05	152	4	1	35.2
	1600	18027	196	1.09	194	2	0	31.7
	1600	14200	94	0.67	93	1	0	29.1
	1600	11528	66	0.57	63	3	0	38.6
$C1_{WT}$	3200	9456	96	1.01	92	4	0	36.5
	3200	17985	231	1.28	230	3	0	38.2
	3200	13021	116	0.89	112	3	1	43.6

Supplementary Table 3. Summary of the single-molecule experiments for wide type C1 with or without EDTA.

Experiment	Pulling speed (nm/s)	Total number	Single-molecule number	Single-molecule ratio (%)	Folded number	Fully unfolded number	Ester bond rupture number	Spring constant (pN/nm)
$C1_{WT}$	1600	4110	69	1.67	58	10	1	35.8
	1600	19590	223	1.14	219	4	0	34.7
	1600	14903	157	1.05	152	4	1	35.2
	1600	18027	196	1.09	194	2	0	31.7
	1600	14200	94	0.67	93	1	0	29.1
	1600	11528	66	0.57	63	3	0	38.6
$C1_{WT}$ (EDTA)	1600	14077	145	1.03	91	19	25	32.6
	1600	14942	143	0.96	77	24	42	36.5
	1600	13472	147	1.08	83	34	30	36.2

Supplementary Table 4. Summary of the single-molecule experiments for all polyproteins with pulling speed of 1600 nm s^{-1}

Experiment	Pulling speed (nm/s)	Total number	Single-molecule number	Single-molecule ratio (%)	Spring constant (pN/nm)
$C1_{WT}$	1600	4110	69	1.67	35.8
	1600	19590	223	1.14	34.7
	1600	14903	157	1.05	35.2
	1600	18027	196	1.09	31.7
	1600	14200	94	0.67	29.1
	1600	11528	66	0.57	38.6
$C1_{T11A}$	1600	34512	946	2.74	40.32
$C1_{CP}$	1600	36881	1114	3.02	37.9
	1600	31357	757	2.41	30.3
C1-df	1600	14652	193	1.32	30.4
C1-df (TCEP)	1600	19856	389	1.96	30.9
$C1_{CP}$-df	1600	23250	277	1.19	28.2
	1600	17612	567	3.21	32.6
$C1_{CP}$-df (TCEP)	1600	23004	754	3.28	26.6
ParV	1600	11292	195	1.73	39.3
mParV	1600	7036	74	1.05	37.8

Supplementary methods

<u>The sequence of individual protein domains/tags</u>

Fgβ

NEEGFFSARGHRPLD

Spytag

AHIVMVDAYKPTK

GB1 (PDB 3MP9)

MDTYKLILNGKTLKGETTTEAVDAATAEKVFKQYANDNGVDGEWTYDDATKTFTVTE

C1$_{WT}$ domain of Cpe0147 (PDB:4MKM & 4NI6)

MPEVKEGTLKTTVAADGVNGSSEKEALVSFENSKDGVDVKDTIDYKDLVANEKYNLTGKLMH
VKDDGSLEEVATKTTEVTAVENGSGQWELDFGNQKLQVGEKYVVFENAESVENLIDTDNNYE
LDTKQVVKHEDKNDKAQTLIVE

Parv (EGV09726 Cna B-type domain protein from Parvimonas sp.)

GEIKTTVMAGGKTSTENEVATLKAKDIEGGVEVSDKITYKGLYPNEKYDVIGKIYEVKDGEL
VNPGHPVSVVNSGDDLKADTTGKGEWTLNFGKLNLEAGKSYVVFETVVSMENVIDTDGDGKP
DKKQELTHEDPNDKSQTFRI

<u>Full polyprotein construct sequences</u>

All constructs were cloned into pET22B or pQE80L vectors containing a 6×HIS (HHHHHH) tag for purification.

SdrG-Cys-6×HIS (PDB:1R17)

MKLGSEQGSNVNHLIKVTDQSITEGYDDSDGIIKAHDAENLIYDVTFEVDDKVKSGDTMTVN
IDKNTVPSDLTDSFAIPKIKDNSGEIIATGTYDNTNKQITYTFTDYVDKYENIKAHLKLTSY
IDKSKVPNNNTKLDVEYKTALSSVNKTITVEYQKPNENRTANLQSMFTNIDTKNHTVEQTIY
INPLRYSAKETNVNISGNGDEGSTIIDDSTIIKVYKVGDNQNLPDSNRIYDYSEYEDVTNDD
YAQLGNNNDVNINFGNIDSPYIIKVISKYDPNKDDYTTIQQTVTMQTTINEYTGEFRTASYD
NTIAFSTSSGQGQGDLPPEKTRSCGTEFAAALEHHHHHH

6×HIS-Cys-SpyCatcher (PDB:4MLI)

MRGSHHHHHHGSCMVDTLSGLSSEQGQSGDMTIEEDSATHIKFSKRDEDGKELAGATMELRD
SSGKTISTWISDGQVKDFYLYPGKYTFVETAAPDGYEVATAITFTVNEQGQVTVNGKATKGD
AHIDRS

Fgβ-linker-(GB1)$_2$-C1$_{WT}$-(GB1)$_2$-Cys-6×HIS

MKLGSNEEGFFSARGHRPLDGSGSGSGSAGTGSGRSMDTYKLILNGKTLKGETTTEAVDAAT
AEKVFKQYANDNGVDGEWTYDDATKTFTVTERSMDTYKLILNGKTLKGETTTEAVDAATAEK
VFKQYANDNGVDGEWTYDDATKTFTVTERSMPEVKEGTLKTTVAADGVNGSSEKEALVSFEN

SKDGVDVKDTIDYKDLVANEKYNLTGKLMHVKDDGSLEEVATKTTEVTAVENGSGQWELDFG
NQKLQVGEKYVVFENAESVENLIDTDNNYELDTKQVVKHEDKNDKAQTLIVERSDTYKLILN
GKTLKGETTTEAVDAATAEKVFKQYANDNGVDGEWTYDDATKTFTVTERSMDTYKLILNGKT
LKGETTTEAVDAATAEKVFKQYANDNGVDGEWTYDDATKTFTVTERSCGTEFAAALEHHHHH
H

6×HIS-Spytag-(GB1-C1$_{WT}$)$_4$-Cys

MRGSHHHHHHGSAHIVMVDAYKPTKRSMDTYKLILNGKTLKGETTTEAVDAATAEKVFKQYA
NDNGVDGEWTYDDATKTFTVTERSMPEVKEGTLKTTVAADGVNGSSEKEALVSFENSKDGVD
VKDTIDYKDLVANEKYNLTGKLMHVKDDGSLEEVATKTTEVTAVENGSGQWELDFGNQKLQV
GEKYVVFENAESVENLIDTDNNYELDTKQVVKHEDKNDKAQTLIVERSMDTYKLILNGKTLK
GETTTEAVDAATAEKVFKQYANDNGVDGEWTYDDATKTFTVTERSMPEVKEGTLKTTVAADG
VNGSSEKEALVSFENSKDGVDVKDTIDYKDLVANEKYNLTGKLMHVKDDGSLEEVATKTTEV
TAVENGSGQWELDFGNQKLQVGEKYVVFENAESVENLIDTDNNYELDTKQVVKHEDKNDKAQ
TLIVERSMDTYKLILNGKTLKGETTTEAVDAATAEKVFKQYANDNGVDGEWTYDDATKTFTV
TERSMPEVKEGTLKTTVAADGVNGSSEKEALVSFENSKDGVDVKDTIDYKDLVANEKYNLTG
KLMHVKDDGSLEEVATKTTEVTAVENGSGQWELDFGNQKLQVGEKYVVFENAESVENLIDTD
NNYELDTKQVVKHEDKNDKAQTLIVERSMDTYKLILNGKTLKGETTTEAVDAATAEKVFKQY
ANDNGVDGEWTYDDATKTFTVTERSMPEVKEGTLKTTVAADGVNGSSEKEALVSFENSKDGV
DVKDTIDYKDLVANEKYNLTGKLMHVKDDGSLEEVATKTTEVTAVENGSGQWELDFGNQKLQ
VGEKYVVFENAESVENLIDTDNNYELDTKQVVKHEDKNDKAQTLIVERSC

Fgβ-linker-(GB1)$_2$-C1$_{T11A}$-(GB1)$_2$-Cys-6×HIS

MKLGSNEEGFFSARGHRPLDGSGSGSGSAGTGSGRSMDTYKLILNGKTLKGETTTEAVDAAT
AEKVFKQYANDNGVDGEWTYDDATKTFTVTERSMDTYKLILNGKTLKGETTTEAVDAATAEK
VFKQYANDNGVDGEWTYDDATKTFTVTERSMPEVKEGTLKATVAADGVNGSSEKEALVSFEN
SKDGVDVKDTIDYKDLVANEKYNLTGKLMHVKDDGSLEEVATKTTEVTAVENGSGQWELDFG
NQKLQVGEKYVVFENAESVENLIDTDNNYELDTKQVVKHEDKNDKAQTLIVERSDTYKLILN
GKTLKGETTTEAVDAATAEKVFKQYANDNGVDGEWTYDDATKTFTVTERSMDTYKLILNGKT
LKGETTTEAVDAATAEKVFKQYANDNGVDGEWTYDDATKTFTVTERSCGTEFAAALEHHHHH
H

Fgβ-linker-(GB1)$_2$-C1$_{CP}$-(GB1)$_2$-Cys-6×HIS

MKLGSNEEGFFSARGHRPLDGSGSGSGSAGTGSGRSMDTYKLILNGKTLKGETTTEAVDAAT
AEKVFKQYANDNGVDGEWTYDDATKTFTVTERSMDTYKLILNGKTLKGETTTEAVDAATAEK
VFKQYANDNGVDGEWTYDDATKTFTVTERSDTKQVVKHEDKNDKAQTLIVEVPGVGVPGVGV
PGEGVPGVGVPGVGVPGVGVPGVGVPGEGVPGVGVPGGLMPEVKEGTLKTTVAADGVNGSSE
KEALVSFENSKDGVDVKDTIDYKDLVANEKYNLTGKLMHVKDDGSLEEVATKTTEVTAVENG
SGQWELDFGNQKLQVGEKYVVFENAESVENLIDTDNNYELRSDTYKLILNGKTLKGETTTEA
VDAATAEKVFKQYANDNGVDGEWTYDDATKTFTVTERSMDTYKLILNGKTLKGETTTEAVDA
ATAEKVFKQYANDNGVDGEWTYDDATKTFTVTERSCGTEFAAALEHHHHHH

Fgβ-linker-(GB1)$_2$-C1-df-(GB1)$_2$-Spytag-6×HIS

MKLGSNEEGFFSARGHRPLDGSGSGSGSAGTGSGRSMDTYKLILNGKTLKGETTTEAVDAAT

AEKVFKQYANDNGVDGEWTYDDATKTFTVTERSMDTYKLILNGKTLKGETTTEAVDAATAEK
VFKQYANDNGVDGEWTYDDATKTFTVTERSMPEVKEGTLKCTVAADGVNGSSEKEALVSFEN
SKDGVDVKDTIDYKDLVANEKYNLTGKLMHVKDDGSLEEVATKTTEVTAVENGSGQWELDFG
NQKLQVGEKYVVFENAESVENLIDTDNNYELDTKQVVKHEDKNDKACTLIVERSDTYKLILN
GKTLKGETTTEAVDAATAEKVFKQYANDNGVDGEWTYDDATKTFTVTERSMDTYKLILNGKT
LKGETTTEAVDAATAEKVFKQYANDNGVDGEWTYDDATKTFTVTERSAHIVMVDAYKPTKRS
GTEFAAALEHHHHHH

Fgβ-linker-(GB1)$_2$-C1$_{CP}$-df-(GB1)$_2$- Spytag -6×HIS

MKLGSNEEGFFSARGHRPLDGSGSGSGSAGTGSGRSMDTYKLILNGKTLKGETTTEAVDAAT
AEKVFKQYANDNGVDGEWTYDDATKTFTVTERSMDTYKLILNGKTLKGETTTEAVDAATAEK
VFKQYANDNGVDGEWTYDDATKTFTVTERSDTKQVVKHEDKNDKACTLIVEVPGVGVPGVGV
PGEGVPGVGVPGVGVPGVGVPGVGVPGEGVPGVGVPGGLMPEVKEGTLKCTVAADGVNGSSE
KEALVSFENSKDGVDVKDTIDYKDLVANEKYNLTGKLMHVKDDGSLEEVATKTTEVTAVENG
SGQWELDFGNQKLQVGEKYVVFENAESVENLIDTDNNYELRSDTYKLILNGKTLKGETTTEA
VDAATAEKVFKQYANDNGVDGEWTYDDATKTFTVTERSMDTYKLILNGKTLKGETTTEAVDA
ATAEKVFKQYANDNGVDGEWTYDDATKTFTVTERSAHIVMVDAYKPTKRSGTEFAAALEHHH
HHH

Fgβ-linker-(GB1)$_2$-C1$_{CP}$-T11A-(GB1)$_2$-Cys-6×HIS

MKLGSNEEGFFSARGHRPLDGSGSGSGSAGTGSGRSMDTYKLILNGKTLKGETTTEAVDAAT
AEKVFKQYANDNGVDGEWTYDDATKTFTVTERSMDTYKLILNGKTLKGETTTEAVDAATAEK
VFKQYANDNGVDGEWTYDDATKTFTVTERSDTKQVVKHEDKNDKAQTLIVEVPGVGVPGVGV
PGEGVPGVGVPGVGVPGVGVPGVGVPGEGVPGVGVPGGLMPEVKEGTLKATVAADGVNGSSE
KEALVSFENSKDGVDVKDTIDYKDLVANEKYNLTGKLMHVKDDGSLEEVATKTTEVTAVENG
SGQWELDFGNQKLQVGEKYVVFENAESVENLIDTDNNYELRSDTYKLILNGKTLKGETTTEA
VDAATAEKVFKQYANDNGVDGEWTYDDATKTFTVTERSMDTYKLILNGKTLKGETTTEAVDA
ATAEKVFKQYANDNGVDGEWTYDDATKTFTVTERSCGTEFAAALEHHHHHH

6×HIS-Spytag-(GB1-Parv)$_2$-Cys

MRGSHHHHHHGSAHIVMVDAYKPTKRSMDTYKLILNGKTLKGETTTEAVDAATAEKVFKQYA
NDNGVDGEWTYDDATKTFTVTERSGEIKTTVMAGGKTSTENEVATLKAKDIEGGVEVSDKIT
YKGLYPNEKYDVIGKIYEVKDGELVNPGHPVSVVNSGDDLKADTTGKGEWTLNFGKLNLEAG
KSYVVFETVVSMENVIDTDGDGKPDKKQELTHEDPNDKSQTFRIRSMDTYKLILNGKTLKGE
TTTEAVDAATAEKVFKQYANDNGVDGEWTYDDATKTFTVTERSGEIKTTVMAGGKTSTENEV
ATLKAKDIEGGVEVSDKITYKGLYPNEKYDVIGKIYEVKDGELVNPGHPVSVVNSGDDLKAD
TTGKGEWTLNFGKLNLEAGKSYVVFETVVSMENVIDTDGDGKPDKKQELTHEDPNDKSQTFR
IRSC

Fgβ-linker- (GB1-mParv)$_2$-Cys-6×HIS

MKLGSNEEGFFSARGHRPLDGSGSGSGSAGTGSGRSMDTYKLILNGKTLKGETTTEAVDAAT
AEKVFKQYANDNGVDGEWTYDDATKTFTVTERSGEIKATVMAGGKTSTENEVATLKAKDIEG
GVEVSDKITYKGLYPNEKYDVIGKIYEVKDGELVNPGHPVSVVNSGDDLKADTTGKGEWTLN
FGKLNLEAGKSYVVFETVVSMENVIDTDGDGKPDKKQELTHEDPNDKSQTFRIRSDTYKLIL

NGKTLKGETTTEAVDAATAEKVFKQYANDNGVDGEWTYDDATKTFTVTERSGEIKATVMAGG
KTSTENEVATLKAKDIEGGVEVSDKITYKGLYPNEKYDVIGKIYEVKDGELVNPGHPVSVVN
SGDDLKADTTGKGEWTLNFGKLNLEAGKSYVVFETVVSMENVIDTDGDGKPDKKQELTHEDP
NDKSQTFRIRSCGTEFAAALEHHHHHH

Reference

1 Latorre, E. *et al.* Active superelasticity in three-dimensional epithelia of controlled shape. *Nature* **563**, 203 (2018).

2 Shen, B.-Q. *et al.* Conjugation site modulates the in vivo stability and therapeutic activity of antibody-drug conjugates. *Nature Biotechnology* **30**, 184-189 (2012).

3 Huang, W. *et al.* Maleimide-thiol adducts stabilized through stretching. *Nature Chemistry* **11**, 310-319 (2019).

4 Shrake, A. & Rupley, J. A. Environment and Exposure to Solvent of Protein Atoms - Lysozyme and Insulin. *Journal of Molecular Biology* **79**, 351-371 (1973).
